# Enhancer Evolution across 20 Mammalian Species

**DOI:** 10.1016/j.cell.2015.01.006

**Published:** 2015-01-29

**Authors:** Diego Villar, Camille Berthelot, Sarah Aldridge, Tim F. Rayner, Margus Lukk, Miguel Pignatelli, Thomas J. Park, Robert Deaville, Jonathan T. Erichsen, Anna J. Jasinska, James M.A. Turner, Mads F. Bertelsen, Elizabeth P. Murchison, Paul Flicek, Duncan T. Odom

**Affiliations:** 1 University of Cambridge, Cancer Research UK Cambridge Institute, Robinson Way, Cambridge, CB2 0RE, UK; 2 European Molecular Biology Laboratory, European Bioinformatics Institute, Wellcome Trust Genome Campus, Hinxton, Cambridge, CB10 1SD, UK; 3 Department of Biological Sciences, University of Illinois at Chicago (UIC), 845 West Taylor Street, Chicago, IL 60607, USA; 4 UK Cetacean Strandings Investigation Programme (CSIP) and Institute of Zoology, Zoological Society of London, Outer Circle, Regent’s Park, London NW1 4RY, UK; 5 School of Optometry and Vision Sciences, Cardiff University, Maindy Road, Cardiff CF24 4HQ, UK; 6 UCLA Center for Neurobehavioral Genetics, 695 Charles E. Young Drive South, Los Angeles, CA 90095, USA; 7 Division of Stem Cell Biology and Developmental Genetics, MRC National Institute for Medical Research, Mill Hill, London NW7 1AA, UK; 8 Center for Zoo and Wild Animal Health, Copenhagen Zoo, Roskildevej 38, DK-2000 Frederiksberg, Denmark; 9 Department of Veterinary Medicine, University of Cambridge, Madingley Road, Cambridge CB3 0ES, UK; 10 Wellcome Trust Sanger Institute, Wellcome Trust Genome Campus, Hinxton, Cambridge, CB10 1SD, UK

## Abstract

The mammalian radiation has corresponded with rapid changes in noncoding regions of the genome, but we lack a comprehensive understanding of regulatory evolution in mammals. Here, we track the evolution of promoters and enhancers active in liver across 20 mammalian species from six diverse orders by profiling genomic enrichment of H3K27 acetylation and H3K4 trimethylation. We report that rapid evolution of enhancers is a universal feature of mammalian genomes. Most of the recently evolved enhancers arise from ancestral DNA exaptation, rather than lineage-specific expansions of repeat elements. In contrast, almost all liver promoters are partially or fully conserved across these species. Our data further reveal that recently evolved enhancers can be associated with genes under positive selection, demonstrating the power of this approach for annotating regulatory adaptations in genomic sequences. These results provide important insight into the functional genetics underpinning mammalian regulatory evolution.

## Highlights

• Rapid enhancer and slow promoter evolution across genomes of 20 mammalian species
• Enhancers are rarely conserved across these mammals
• Recently evolved enhancers dominate mammalian regulatory landscapes
• Unbiased mapping links candidate enhancers with lineage-specific positive selection

## Graphical Abstract

**Figure:**
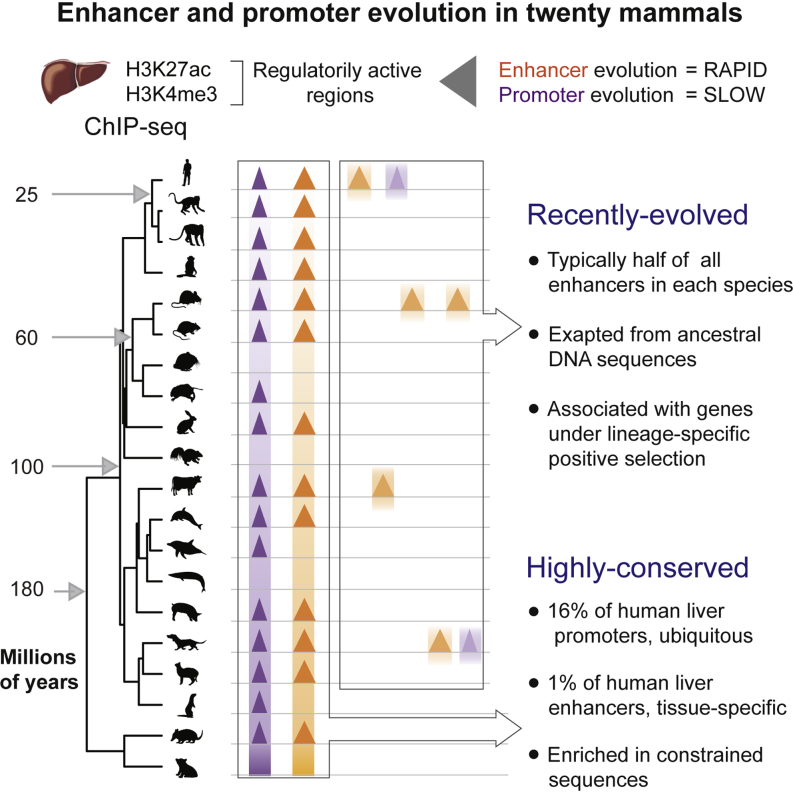

## Teaser

Comparative functional genomic analysis in 20 mammalian species reveals distinct features for the evolution of enhancers, in comparison to those of promoters, across 180 million years.

## Introduction

Most mammalian genes are controlled by collections of enhancer regions, often located tens to hundreds of kilobases away from transcription start sites. Recent studies comparing key selected mammals (Cotney et al., 2013; Xiao et al., 2012) have indicated that enhancers may change rapidly during evolution (Degner et al., 2012; Shibata et al., 2012), particularly when compared with evolutionarily stable gene expression patterns (Brawand et al., 2011; Chan et al., 2009; Merkin et al., 2012). Given that most phenotypic differences are hypothesized to largely result from regulatory differences between mammals, it is of profound importance to understand the mechanisms driving enhancer evolution (Villar et al., 2014; Wray, 2007).

Both conserved and recently evolved enhancer sequences have been shown to have important phenotypic consequences. Highly conserved enhancer sequences can regulate fundamental processes, such as embryonic development, and this property has been used to screen for functional regulatory elements (Pennacchio et al., 2006). However, sequence-level changes in enhancer elements can also underlie evolutionary differences between species (Hare et al., 2008; Ludwig et al., 2005), as has now been demonstrated across many organisms (Arnold et al., 2014; Cotney et al., 2013; Degner et al., 2012; McLean et al., 2011; Shibata et al., 2012).

Approaches comparing vertebrate genome sequences, such as those employing 29 mammals, have revealed regulatory regions under sequence constraint (Lindblad-Toh et al., 2011). However, this approach is limited in resolving tissue-specific deployment or regulatory activity directed by small sequence changes, particularly as may be predicted for rapidly evolving enhancer regions (however, see Pollard et al., 2006; Prabhakar et al., 2006). Comparative analysis of mammalian genomes can indicate protein sequence adaptations in particular species or lineages, and infer which coding regions are under positive selection. In contrast, complementary experimental efforts are currently lacking to functionally annotate the many recently sequenced mammalian genomes.

Experimental tools can now empirically identify regulatorily active DNA across entire mammalian genomes. Enhancers can be identified by mapping regions enriched for acetylated lysine 27 on histone H3 (H3K27ac) via chromatin immunoprecipitation followed by high-throughput sequencing (ChIP-seq) (Creyghton et al., 2010). Similarly, active gene promoters can be identified as containing both H3K27ac and trimethylated lysine 4 of histone H3 (H3K4me3), which marks sites of transcription initiation (Cain et al., 2011; Santos-Rosa et al., 2002). The usefulness of this approach to map regulatory activity genome-wide has been recently underscored by analysis of H3K27ac dynamics across organ development in mouse (Nord et al., 2013). This study found that most H3K27ac developmental variation occurs distally to transcription start sites and within predicted enhancer elements, most of which could be validated experimentally.

Over 20 sequenced mammalian genomes have been integrated into inter-species alignments within Ensembl (Flicek et al., 2014). Exploiting this computational infrastructure (and related resources in *Drosophila*; Kim et al., 2009), recent studies have dissected how transcription factor (TF) binding has evolved (He et al., 2011; Paris et al., 2013; Schmidt et al., 2010; Stefflova et al., 2013). In addition, enhancer and promoter evolution have been investigated using sets of mammals, where H3K27ac levels have been characterized across tissues and developmental states as a proxy for enhancer function and developmental or tissue-specific gene expression (Cotney et al., 2013; Nord et al., 2013; Xiao et al., 2012).

Here, we report the results of empirically mapping promoter and enhancer evolution across 20 mammals chosen to span the breadth and depth of the class Mammalia, including previously uncharacterized species such as cetaceans and naked mole rat. Our analyses have revealed the tempo and mechanisms underlying enhancer evolution across over 180 million years of mammalian radiation.

## Results

### Profiling Promoter and Enhancer Regulatory Evolution in Mammalian Liver

**Figure 1:**
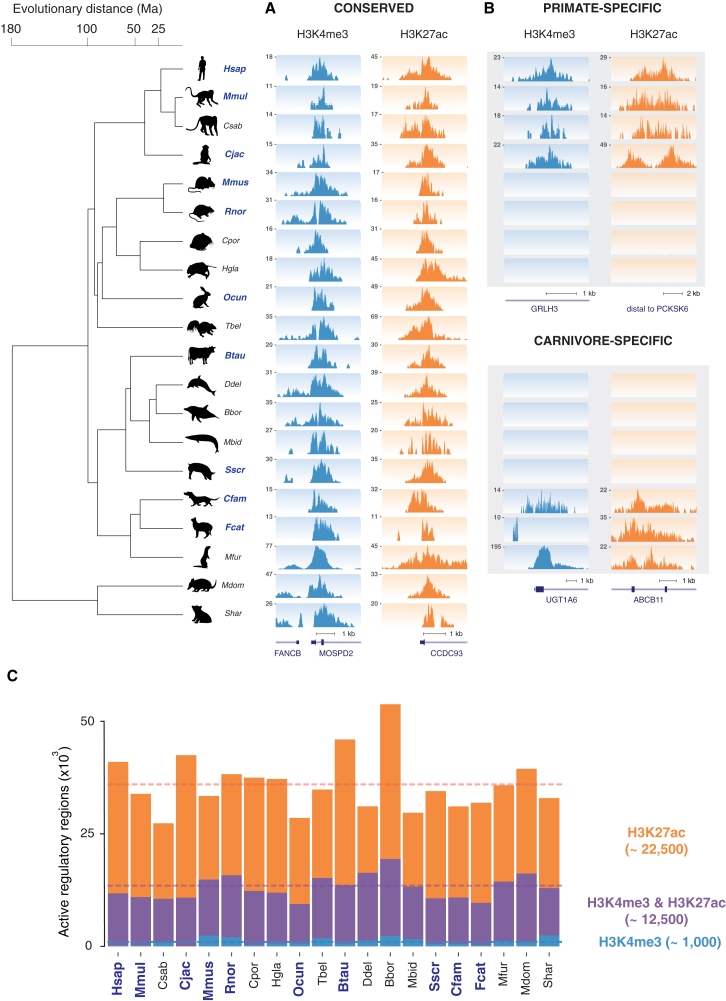
In Vivo Regulatory Activity Assessed in Livers from 20 Mammals (A and B) Phylogenetic relationships and species divergences are represented by an evolutionary tree, which includes 18 placental species (in four orders) and 2 marsupial species (in two orders). In liver isolated from each species, enhancer activity was globally mapped by identifying genomic regions enriched for acetylation of H3K27 (H3K27ac), and transcription initiation was mapped by identifying genomic regions enriched for tri-methylation of H3K4 (H3K4me3). Shown are examples of regulatory regions active: (A) across all 20 species (MOSPD2 and CCDC93 loci), and (B) active only in primates (GRLH3 and PCKSK8, top) or active only in carnivores (UGT1A6 and ABCB11, bottom). For order-specific regulatory regions, data from some species are not shown for conciseness. (C) In liver, a typical mammalian genome contains ∼22,500 enhancers enriched for only H3K27ac; ∼12,500 promoters enriched for both H3K27ac and H3K4me3 and ∼1,000 containing only H3K4me3. Highest quality genomes incorporated into the EPO multiple alignment are labeled in blue (Experimental Procedures). See also Figures S1 and S2 and Tables S1 and S2.

We mapped the active promoter and enhancer elements in liver as a representative adult somatic tissue from 20 species of mammals (Figure 1). Study species were selected using three criteria: (1) to capture a substantial fraction of the mammalian phylogenetic tree, (2) to profile the major placental orders in a combination of intra- (6–40 Ma) and inter-lineage (100–180 Ma) evolutionary distances, and (3) to extend our understanding of regulatory evolution to previously uncharacterized mammals whose phenotypes are highly divergent, such as cetaceans, naked mole rat, and Tasmanian devil. Liver from almost all study species was profiled in biological replicates from two or more individuals, except for Sei Whale *(Balaenoptera borealis)*, where only one individual’s tissue was available; and for dolphin, for which we combined data from two closely related dolphin species (*Delphinus delphis* and *Lagenorhynchus albirostris*) where a single individual from each species was profiled (Tables S1 and S2, Experimental Procedures).

**Figure S1:**
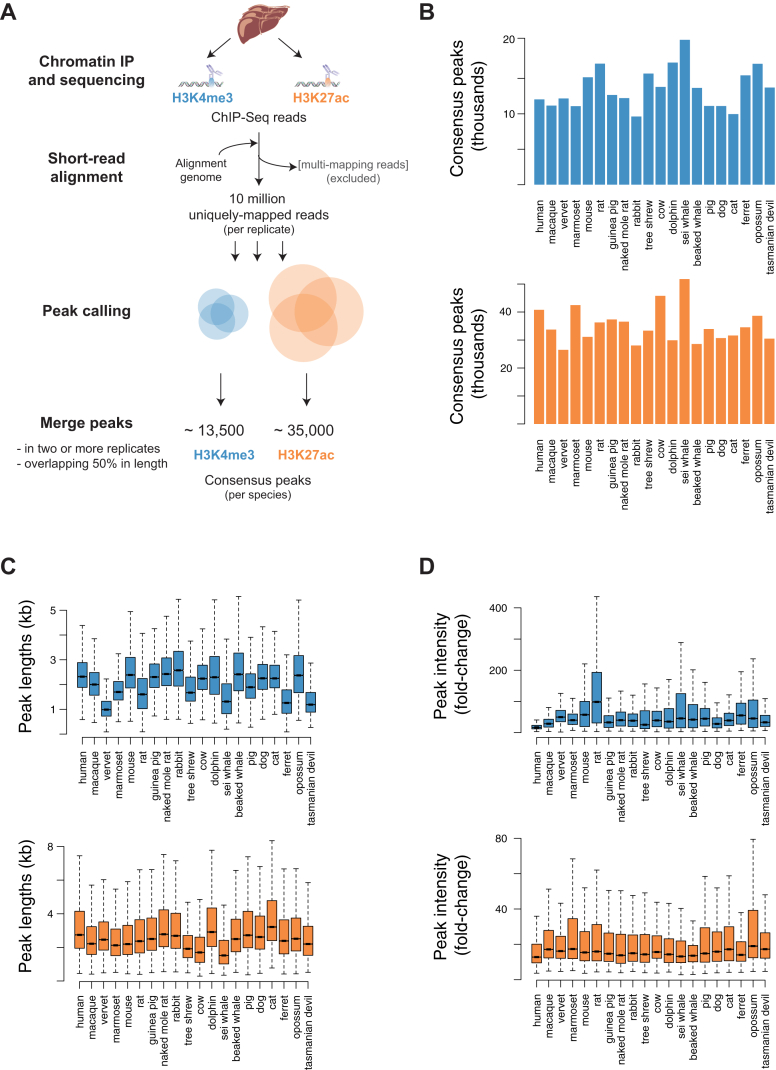
Analysis Workflow and Quality Control of H3K4me3 and H3K27ac ChIP-Seq in 20 Mammals, Related to Figure 1 (A) Short-read alignment and peak calling workflow (see also Extended Experimental Procedures) (B) Numbers of consensus peaks identified for H3K4me3 (blue) or H3K27ac (orange) in each species’ liver tissue. (C) Length distributions of consensus H3K4me3 (blue) or H3K27ac (orange) peaks are represented as boxplots for each species. (D) Peak intensity distributions are represented as boxplots for each species’ data (H3K4me3, blue; H3K27ac, orange). Peak intensities correspond to average fold enrichment values over total input DNA across biological replicates (see Extended Experimental Procedures).

We quantified using ChIP-seq the genome-wide occurrence of two key histone marks widely used to profile promoters and enhancers: H3K4me3 and H3K27ac (Figure 1) (Creyghton et al., 2010; Santos-Rosa et al., 2002). We identified regions enriched for these histone marks within each mammalian liver genome using only biologically reproducible peaks present in two or more replicates (Figure S1, Experimental Procedures).

**Figure S2:**
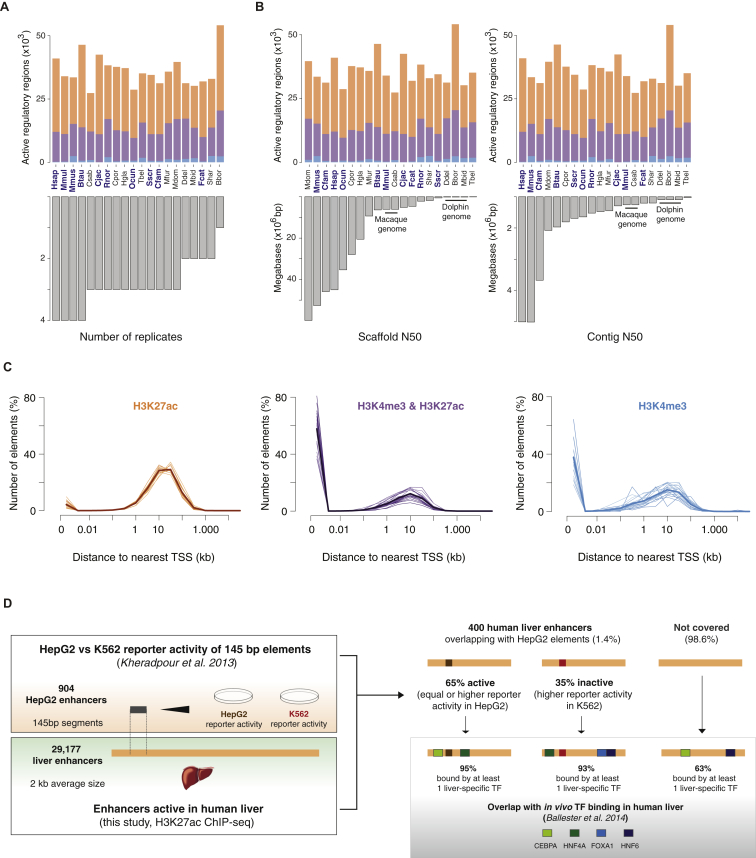
Quality Control of Experimental Promoter and Enhancer Definition, Related to Figure 1 (A) Numbers of experimentally identified promoters (H3K4me3&H3K27ac, purple; H3K4me3, blue) and enhancers (H3K27ac, orange) per species are represented as stacked barplots in the upper plot, ordered by decreasing number of biological replicates used for each species (lower plot). Except for Bbor (*Balaenoptera borealis*), where a single replicate was used, the number of biological replicates has little influence on the number of active regulatory regions identified per species. (B) As in (A), but numbers of promoters/enhancers in each species are now ordered by decreasing scaffold or contig N50 values, both indicative of genome assembly quality. Species highlighted in blue correspond to genomes in the EPO multiple alignment, considered to be the highest-quality reference genomes. Assembly qualities do not appear to influence experimental variation in the number of promoters or enhancers identified in each species. (C) The distribution of distances to the nearest transcriptional start site (TSS) was calculated for all experimentally identified regions in each species’ data (thin lines). Bolded lines represent the average distance distribution across all species for H3K4me3 (blue), H3K4me3&H3K27ac (purple) and H3K27ac (orange) elements. In agreement with their categorisation as enhancer elements, in all species most H3K27ac locations are distal to coding regions. Both H3K4me3 and H3K4me3&H3K27ac elements are largely located close to annotated TSSs consistent with being proximal promoters. The minority of distal elements marked by H3K4me3 or H3K4me3&H3K27ac may correspond to unannotated transcripts; further, the latter may also act as enhancers (Kim et al., 2010). (D) H3K27ac-defined enhancers enrich for regulatory activity: Human liver enhancers identified in this study through H3K27ac ChIP-seq (bottom inset) were overlapped with 145 bp sequence elements assayed for reporter activity in human liver carcinoma (HepG2) and human erythroleukemia cells (K562) (top inset; Kheradpour et al., 2013). These correspond to enhancer candidates identified in HepG2 cells and containing motifs for liver-specific transcription factors. Four hundred human liver enhancers contained at least one 145 bp segment (1.1 segments per enhancer on average). 65% of these enhancers were active based on the reporter activity of the assayed segments, which displayed higher activity in HepG2 compared to K562 cells, or equal activity in both cell lines. The remaining 35% human liver enhancers overlapped segments having higher activity in K562 cells, and were thus classified as inactive in HepG2 cells. Grey inset: Human liver enhancers identified in this study were overlapped with in vivo binding locations for four liver-specific transcription factors, as reported independently in human liver samples (Ballester et al., 2014). Among the 400 enhancers containing segments assayed in Kheradpour et al., 93%–95% of them were bound by at least one liver-specific TF, regardless of the reporter activity of their overlapping segments. This suggests that in cases where the overlapping segment was inactive in the reporter assay, the corresponding enhancer may harbor regulatory activity outside the interrogated sequence. Across all liver enhancers in human, 63% are bound by at least one of the four liver-specific transcription factors, in line with previous estimates of functional enhancer activity in H3K27ac-marked regions (Nord et al., 2013).

A total of 30–45,000 regions per species were enriched in liver, and these separated into H3K27ac, H3K4me3&H3K27ac, and H3K4me3-marked elements (Figures 1C and S1). Our analyses were robust to variability in the genome assembly quality and sample preparation (Experimental Procedures and Figure S2). We confirmed that H3K4me3 often co-occupied the genome with H3K27ac (Heintzman et al., 2009; Zhu et al., 2013), and that most H3K4me3-positive regions occur at transcriptional start sites (Cain et al., 2011; Santos-Rosa et al., 2002), regardless of their H3K27ac enrichment (see Experimental Procedures). In contrast, regions enriched for H3K27ac often were not enriched for H3K4me3, and these often located far from transcriptional start sites (Figure S2).

The regions we identify as enhancers strongly enrich for regulatory activity in liver, consistent with numerous prior studies (Cotney et al., 2013; Creyghton et al., 2010; Nord et al., 2013; Zhu et al., 2013). For over 400 of our human liver enhancers (typically 2 kb in length), the transgenic activities of overlapping 145 bp segments were assayed in liver cancer cells (Kheradpour et al., 2013) (Figure S2). Although each human liver enhancer was on average represented by only a single small sequence element, capturing less than 10% of the enhancer length, over 65% showed activity in transgenic assays in a cancer cell line. Furthermore, over 90% of the enhancers not active in transgenic assays were nevertheless bound in human liver by at least one liver-specific TF (Ballester et al., 2014). In sum, this analysis suggests a sizable majority of our empirically determined enhancers are regulatorily active.

Our data newly demonstrates that the known interplay of H3K4me3 and H3K27ac creates a genomic regulatory landscape that is a uniform feature across mammals (and likely across eumetazoans; Schwaiger et al., 2014). In adult liver, a typical mammalian genome contains on average 12,500 H3K4me3 locations (representing active promoter elements) and 22,500 H3K27ac-enriched regions (representing active enhancers).

### Enhancer Evolution Is Appreciably More Rapid Than Proximal Promoter Evolution

**Figure 2:**
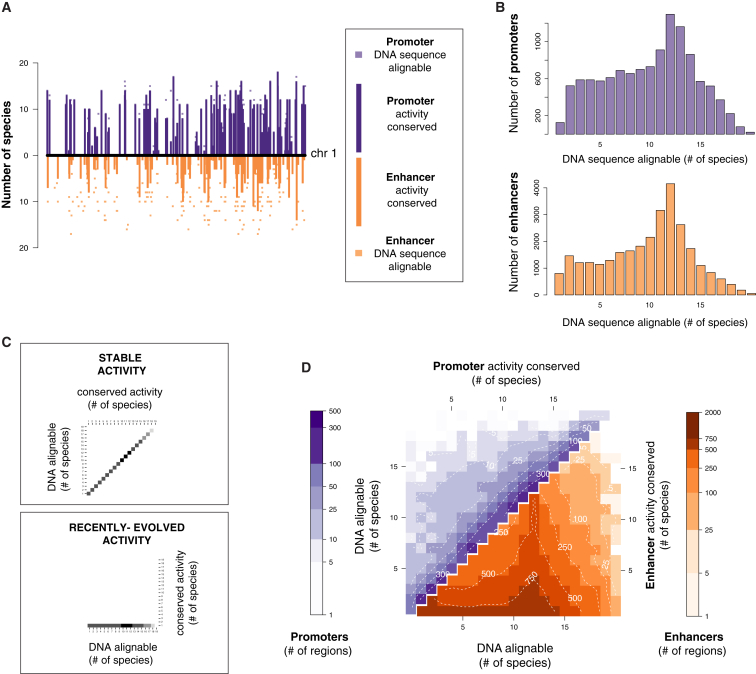
Enhancers Evolve Rapidly; Promoters Are Highly Conserved (A) For a representative 10 MB region on human chromosome 1, the bar chart on the y axis represents the number of species in which enhancer and promoter elements were active (promoters: top, purple; enhancers: bottom, orange). Squares indicate the number of species where the sequence underlying the active promoter or enhancer was alignable. (B) The DNA sequences underlying proximal promoters and the DNA sequences underlying enhancers can be aligned to similar numbers of species, suggesting that differences in apparent conservation of activity are not due to differences in alignability. (C) Schematic diagram showing how the conservation of regulatory activity versus DNA alignability across 20 species of mammals can reveal (top) where DNA function and DNA sequence orthology closely correspond, indicating ancestral activity, and (*bottom*) where pre-existing DNA sequences have been exapted within specific lineages or species, indicating recently evolved activity. (D) Our data revealed that if the DNA underlying a human-identified proximal promoter region (purple) can be aligned with an orthologous sequence in another species, then promoter activity is very often present as well (heatmap enrichment concentrated on the diagonal of the plot). In contrast, most enhancer regions (orange) are rapidly evolving within older DNA sequences, reflected in increased heatmap enrichment toward the lower x axis. Color scales and dashed contour lines indicate absolute numbers of active promoter or enhancer regions (logarithmic scale). See also Figure S3.

**Figure S3:**
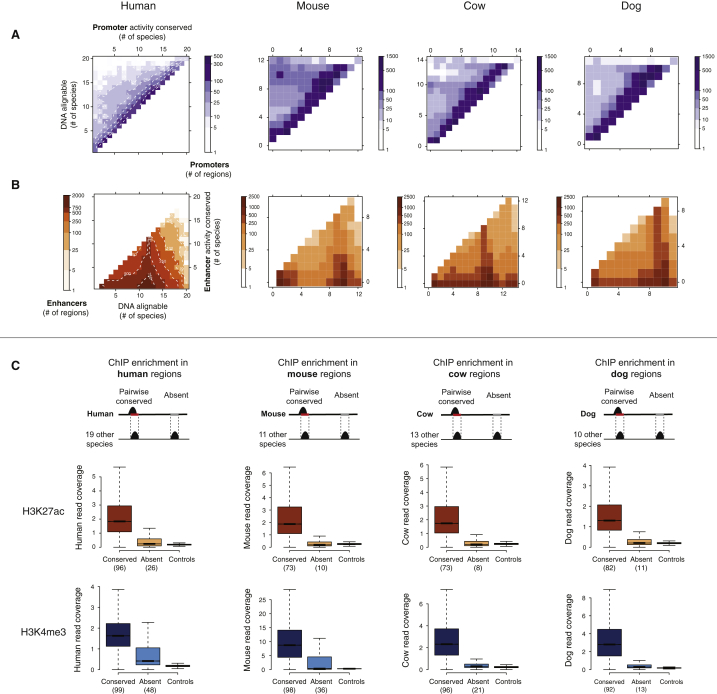
Conservation of Activity Assessed in Four Representative Mammals, Related to Figure 2 (A and B) Regardless of the species used as a reference, liver promoter activity (A) is usually conserved in most species where an orthologous region (i.e., DNA alignable) can be found. Conversely, enhancer activity (B) evolves rapidly and is typically conserved across few species, although the DNA sequences underlying enhancers can usually be aligned across a larger number of mammals than those with enhancer activity. (C) Assessment of false negatives in pairwise species comparisons: raw sequence read counts were calculated within a reference species (for instance, human, far left diagram) at sites that are orthologous to active regions in other species in the dataset. These sites can either be conserved, if the region is active in human also; or non-conserved (“Absent”), if it is not detected as active in human. Some absent regions may contain promoter or enhancer activity that falls below either the significance threshold or the reproducibility criteria used for peak calling (Figure S1), and thus would represent false negatives. Boxplots below each diagram represent distributions of read coverage at conserved and absent regions, using data from four different reference species (human, mouse, cow, and dog) for each histone mark. For each region, a single coverage value was calculated, corresponding to the average coverage over replicates after normalization for total library size. Read coverage at these sites in the total DNA controls (no antibody) was used as a control distribution. Numbers under each “Conserved” or “Absent” box indicate the percentage of regions with read coverage in the upper tail of the control distribution (> mean + 1.96^∗^sd). In most cases, coverage at absent sites is very similar to the control and markedly different from conserved regions, indicating low false negative rates. A proportion of human and mouse H3K4me3 absent sites display higher read coverage than the control, suggesting that conservation of promoter activity may be even higher than reported in our main analyses.

We used our genome-wide mapping data in livers from 20 mammals to obtain an empirical and quantitative understanding of evolutionary stability of promoters and enhancers (Figure 2 and Figure S3).

Most non-coding regions in the human genome cannot be mapped across 20 mammals, in large part because the genome structure and regulatory content of complex eukaryotes evolve rapidly (Lynch et al., 2011). We defined the maximum detectable conservation of activity as the number of species in which the DNA could be aligned (Figure 2A). For example, if enhancer activity is highly conserved, then this activity would be detected in all species where the underlying DNA was alignable. In contrast, low conservation would be characterized by the underlying DNA remaining alignable across many species, but without sharing of enhancer activity. Such low conservation could be a signature of rapid functional evolution or, alternatively, functional neutrality.

Collectively, the DNA sequences used as promoters and the DNA sequences used as enhancers in liver show only slight differences in their alignability across the study species (Figure 2B). This alignability shows a marked increase at approximately 11–13 species, reflecting the contribution to the multiple alignments of the ten highest-quality genomes (Experimental Procedures).

The conservation of active liver promoters tracked remarkably closely with the alignability of the underlying DNA, indicating evolutionarily stable promoter activity (Figure 2C, upper left triangle). In other words, the transcription initiation sites driving gene expression in liver are highly conserved.

We performed a similar analysis for enhancers. Our data reveal that rapid enhancer evolution, often involving exaptation of ancestral DNA, is active and widespread across all the mammalian clades in our study (Figure 2D, orange, and Figure S3), as has been reported in primates (Cotney et al., 2013). Furthermore, the ten highest-quality placental genome sequences contained thousands of cross-alignable regions where enhancer activity was shared in many, but not all, species. These regions are liver enhancers that were likely present in the common placental ancestor and have partially degraded along some lineages. In contrast to promoter sites, enhancer locations evolve rapidly, and comparatively few are deeply conserved (see below). Control analyses show that while promoter conservation may be under-estimated, this is not the case for enhancers (Figure S3).

**Figure 3:**
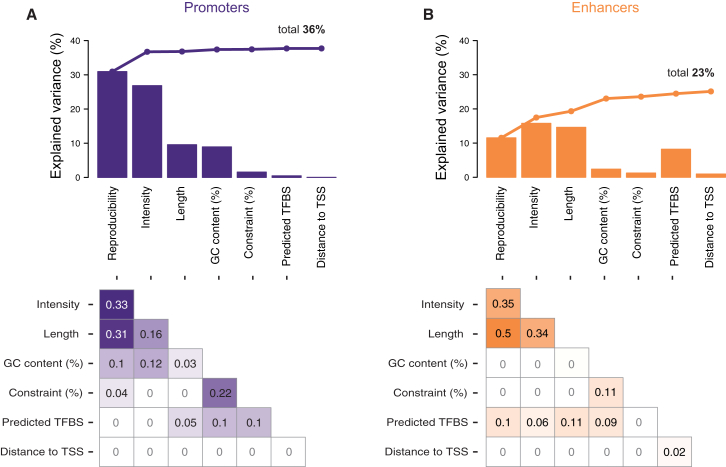
Features Contributing to Conservation of Promoter and Enhancer Activity Identified in Human Liver (A) For all human proximal promoters active in liver, the depth of conservation was correlated with experimental features (reproducibility, peak intensity, peak length, distance to nearest transcription start site) as well as underlying genomic features (GC content, sequence constraint, TF binding sites). Each feature in isolation explained a significant fraction of the variance in conservation of promoter activity (e.g., peak length explained 10%). The fraction explained by the features in combination, when added left to right using multiple regression analysis, are plotted as a line above, in sum totaling 36%. The increases in explained variance with the addition of each feature are attenuated due to strong inter-correlation of features, quantified in the bottom panel as R^2^ values between features (Experimental Procedures). (B) The same analysis was performed for human liver enhancers, where experimental and genomic features together explained a more modest fraction (23%) of the conservation of enhancer activity in other species.

We asked whether the conservation of liver promoters and enhancers is associated with underlying sequence features (e.g., TF binding sequences, %GC content, sequence constraint), experimental features (reproducibility, occupancy level/intensity, length), or some combination (Figure 3). The best predictor of conservation in promoter regions is the reproducibility and strength of enrichment of H3K4me3 and H3K27ac, with the length of the histone-modified domain and GC content as separate, modest contributors. Thus, experimental features are stronger indicators of the conservation of regulatory activity, and underlying sequence features contribute less to promoter stability. In contrast, the presence of TF binding sites can explain a modest fraction of the conservation of enhancer activity. Nevertheless, as with promoters, the enrichment reproducibility and intensity of signal is the primary predictor of conservation. Collectively, no combination of sequence- and experimental-based features could potentially explain more than a third of the variance in conservation of regulatory activity.

Overall, our data reveal that promoter activity in a representative somatic tissue is highly constrained across mammalian space. In contrast, enhancer evolution is rapid and widespread. Neither enhancer nor promoter activity conservation can be explained purely by underlying sequence elements.

### Quantifying the Divergence Rates of Enhancers, Promoters, and TF Binding in a Cross-Section of Mammals

The divergence rate of sequence-specific transcription factor binding (Stefflova et al., 2013) and the extent of regulatory evolution (Cotney et al., 2013; Shibata et al., 2012; Xiao et al., 2012) has been estimated using matched experiments from the same tissues in subsets of typically three to five mammals within a single order. We took a similar approach to calculate how rapidly enhancers and promoters active in liver evolve across 20 mammals.

**Figure S4:**
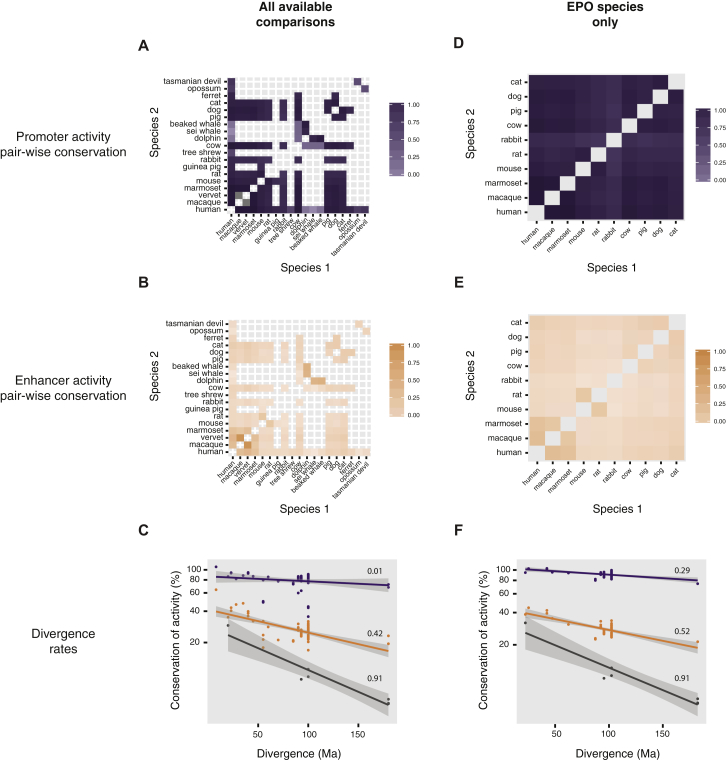
Experimentally Determined Rates of Promoter and Enhancer Evolution across Mammals, Related to Figure 4 (A–F) The average fraction of regions with pairwise conserved activity for promoters (purple) or enhancers (orange) represented as heatmaps, as measured for: (1) all available comparisons in the dataset (A and B), using the 13 eutherian mammals Ensembl EPO multiple alignment where possible and ad hoc LastZ pairwise alignments otherwise (see Extended Experimental Procedures). (2) only species in the 13 eutherian mammals Ensembl. EPO multiple alignment (D and E), corresponding to the higher-quality reference genomes in the dataset. The choice of species and alignments had no significant influence on the rates, as calculated by an exponential decay fit to either set of comparisons. Percent conservation (y axis) is shown in logarithmic scale, and numbers above each dataset represent R^2^ values for the exponential decay fit (C and F). The regressions (solid lines) in (C) were used to calculate the estimated half-lives and mean lifetimes in Figure 4 (promoters: half-life 939 Ma [641-1760], mean lifetime 1355 Ma [924-2539]; enhancers: half-life 296 Ma [231-408]; mean lifetime 427 Ma [334-589]; CEBPA binding sites: half-life 144 Ma [103-237]; mean lifetime 207 Ma [148-342]). Numbers in square brackets indicate 95% confidence intervals for each value.

We first identified, by pairwise analysis of all 20 species, whether regions called as enhancers and promoters were present in the same location between two mammalian genomes (Experimental Procedures, Figure S4). Because this analysis does not use human as the primary reference genome, we could generate multiple independent estimates of how evolutionarily stable enhancers and promoters were for comparable divergence distances. Further, divergence rates could be estimated for evolutionary distances not available from a human-centric analysis. For instance, our data provided multiple comparisons of species separated by 40 to 100 Ma using mouse, cow, or dog as reference that could not be obtained using a human-centric approach (Figure 1).

**Figure 4:**
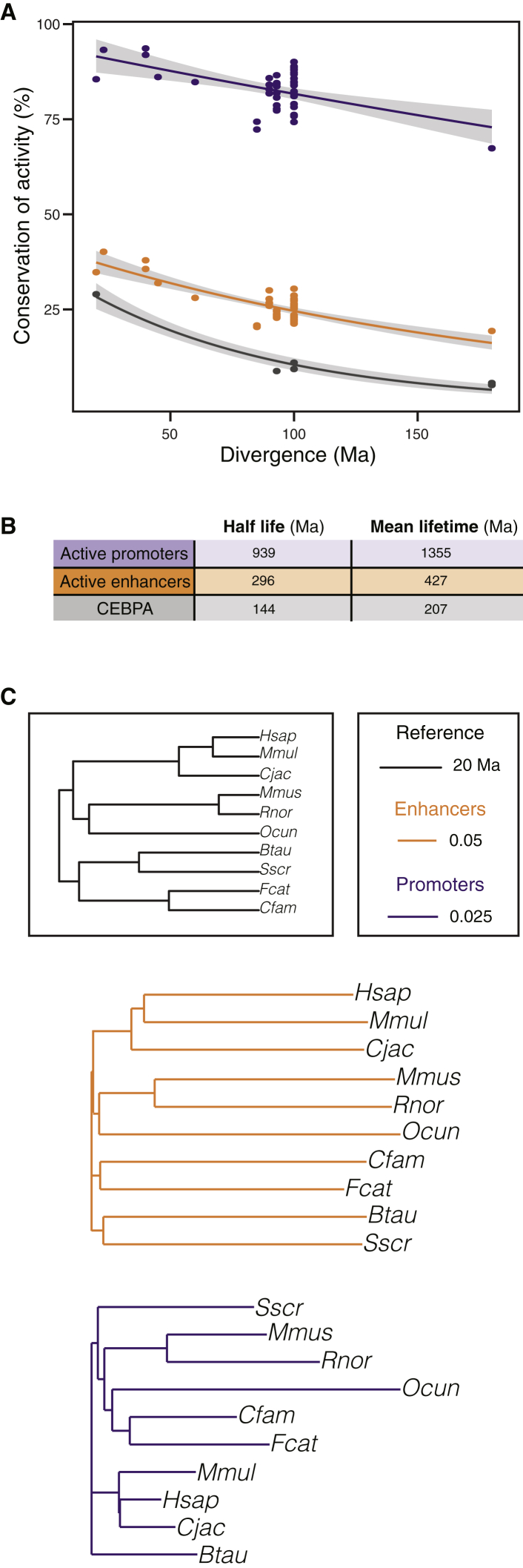
Empirically Determined Rates of Promoter, Enhancer, and TF Binding Divergence in Liver across 180 Million Years of Mammalian Evolution (A) For promoters (purple), enhancers (orange), and TF binding sites (CEBPA, black), the fraction of ChIP-seq peaks present at the orthologous location between pairs of mammals are shown as a function of evolutionary distance. Solid lines represent an exponential decay fit, surrounded by gray shading of a 95% confidence interval (Experimental Procedures). For liver promoters and enhancers, we used data from the ten highest-quality placental genomes, while CEBPA data have been previously reported (Schmidt et al., 2010). (B) Comparative half-lives and mean-lifetimes (in million years) for active promoters, enhancers and CEBPA transcription factor binding locations, as calculated from the exponential decay fits in (A). (C) Neighbor-joining phylogenetic trees based on pairwise conservation levels of enhancer and promoter activity, as measured in (A). Enhancer evolution (orange) recapitulates the known relationships among the studied mammals (black). The low divergence of promoter activity is insufficient to resolve the phylogenetic groups (purple). See also Figure S4.

Inter-species conservation of promoters and enhancers could be plausibly described as a function of time-of-divergence by fitting an exponential decay curve (Experimental Procedures). In liver, promoters diverged at a slower rate than did either enhancers or TF bound regions (Figure 4 and Figure S4). Interestingly, promoters’ half-lives are comparable to protein-coding genes’ half-lives, at over a billion years (Rands et al., 2014). The higher stability of promoters versus enhancers could be due in part to the intimate functional connection promoters have with the first exon of protein coding genes, which are highly stable features of vertebrate genomes (Lindblad-Toh et al., 2011). Our results are consistent with a model where the increased size and sequence heterogeneity of regions with promoter or enhancer activity could buffer evolutionary changes more robustly than can site-specific TF binding alone (Cotney et al., 2013; Shibata et al., 2012; Xiao et al., 2012).

### Highly Conserved Regulatory Regions Are Largely Proximal Promoters

Our mapping of liver enhancer and promoter evolution using mammals spanning both intra-order (6–40 Ma) and inter-order (80–180 Ma) divergence times permits the dissection of conserved (and recently evolved, see below) regulatory regions.

**Figure 5:**
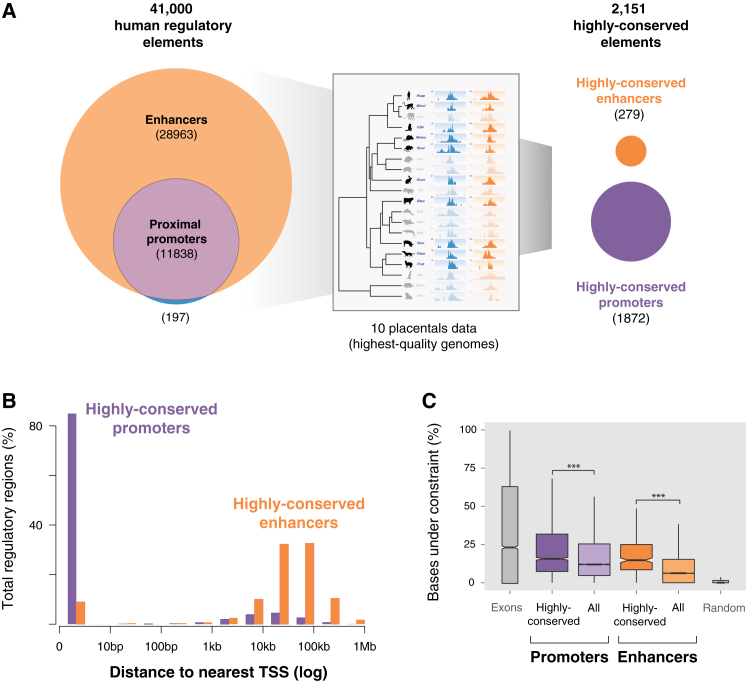
Most Highly Conserved Liver Regulatory Regions Are Proximal Promoters (A) The ∼41,000 regulatorily active regions in human liver are shown on the left panel (enhancers: orange; promoters: purple). The regulatory elements with conserved activity in the ten placental species with highest quality genomes (boxed inset) were determined by cross-species comparison (Experimental Procedures), identifying approximately 300 enhancers and 1,800 promoters (labeled as highly conserved, right panel). (B) Almost all highly conserved promoter regions (purple) are located at transcription start sites as expected, whereas conserved enhancer regions (orange) are typically tens to hundreds of kilobases from the nearest gene. (C) Regions of highly conserved enhancer and promoter activity show a corresponding, but modest, increase in selective constraint in their underlying DNA sequence. The distribution of the fraction of bases under constraint in each region within each category is shown as a box-plot, with human exons and randomly selected regions shown for comparison (Experimental Procedures).^∗∗∗^ indicates p value < 2 × 10^−16^, Wilcoxon test. See also Figures S5 and S6 and Tables S3, S6, and S7.

We first quantified how many regions showed strong conservation of activity by defining regions as *highly conserved* if regulatory activity was present in (at a minimum) all ten of the highest-quality placental genomes (Figure 5A). A total of 2,151 genomic regions appeared highly conserved by these criteria, representing 5% of all human regions active in liver. The existence of over 2,000 highly conserved regions is greater than expected by chance (p value < 1 × 10^−4^, random permutation test, Experimental Procedures).

Highly conserved regions were classified as promoters or enhancers based on their consensus histone mark enrichment across all 20 mammals (Experimental Procedures). Of these 2,151 highly conserved regulatory regions, 1,871 elements (87%) were enriched for both H3K27ac and H3K4me3, consistent with acting as promoters (Santos-Rosa et al., 2002).The vast majority of highly conserved promoters occupied the transcription start sites of genes (Figure 5B). On the other hand, a subset of 279 regions showed enrichment only for H3K27ac occupancy, consistent with acting as enhancers (Creyghton et al., 2010). Most highly conserved enhancers were tens to hundreds of kilobases away from the nearest gene (Figure 5B). The single region uniformly enriched across placentals for only H3K4me3 is not shown.

In human liver, there are 11,838 promoter regions enriched for both H3K27ac and H3K4me3, and 28,963 enhancer regions containing only H3K27ac. Although nearly three times as common as promoters, the activity of only 1% of these enhancers is highly conserved. In contrast, the activity of 16% of promoters is highly conserved (Figure 5A).

**Figure S5:**
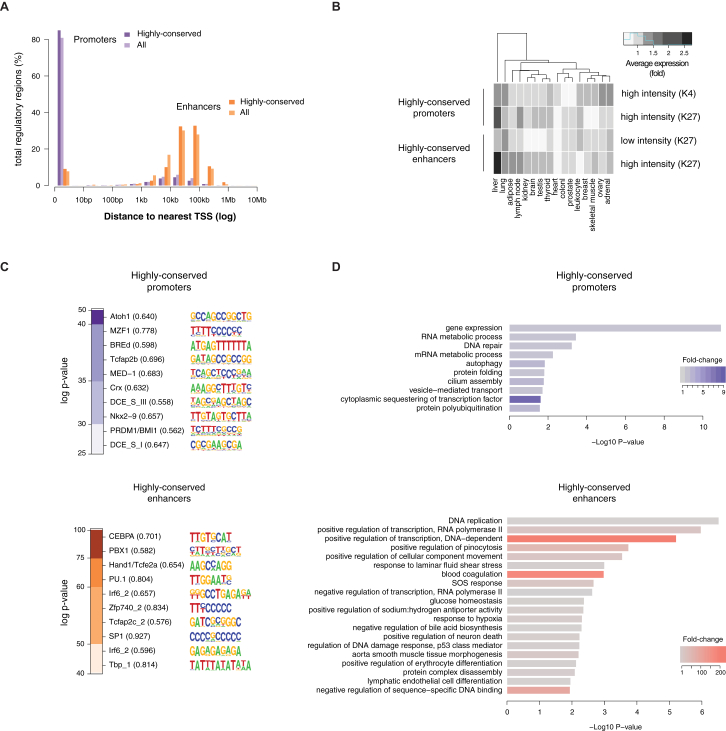
Additional Properties of Highly Conserved Promoters and Enhancers, Related to Figure 5 (A) The distribution of distances to the nearest TSS is almost identical between highly conserved promoters or enhancers (darker purple and orange, respectively) and all experimentally identified promoters/enhancers in human (lighter purple and orange bars). (B) Average expression of genes associated to highly conserved promoters and enhancers across a panel of 16 human tissues (Petryszak et al., 2014). Highly conserved enhancers are associated with genes showing a higher average expression in liver, especially for the top 50% H3K27ac intensities (“high intensity (K27)”). Conversely, highly conserved promoters are largely associated with ubiquitously expressed genes, although promoters with high H3K27ac intensity also associate with high liver gene expression. Expression profiles for genes associated with all promoters and enhancers identified in human were used as background to normalize expression values (see Extended Experimental Procedures). (C) Sequence motifs specifically enriched in highly conserved promoters and enhancers, using all experimentally identified promoters or enhancers in human as a background control. The ten most-enriched motifs are shown, and enrichment p values are represented as heatmaps (logarithmic scale). (D) Gene ontology annotations for biological processes enriched near highly conserved promoters and enhancers. Liver-related annotations such as blood coagulation, glucose homeostasis or bile acid biosynthesis are found for highly conserved enhancers, in line with their association to liver-specific genes.

Three independent lines of evidence support the functionality of the sequences we identify as highly conserved regulatory regions in liver. First, all show enhanced sequence constraint (Figure 5C). Second, genes near highly conserved enhancers are strongly enriched for liver-specific functions, and genes near conserved proximal promoters are enriched for house-keeping functions (Figure S5, Tables S3 and S6) (Forrest et al., 2014). Third, highly conserved enhancers are enriched for TF binding motifs for liver-specific regulators such as CEBPA and PBX1, whereas highly conserved proximal promoters appear dominated by transcriptional initiation regulatory sequences (Figure S5, Table S7).

In sum, in adult mammals comparatively few enhancers are evolutionarily stable. In contrast, a substantial fraction of the proximal promoters found in human liver appear to be highly conserved across mammals.

### Recently Evolved Regulatory Activity Is Pervasive in Mammals

Even for proximal promoters, the number of highly conserved regulatory elements active in liver is a small fraction of the total number experimentally identified in any single species (Figure 5 and Table S4). We sought to identify and analyze the molecular features of more recently evolved regulatory regions.

**Figure 6:**
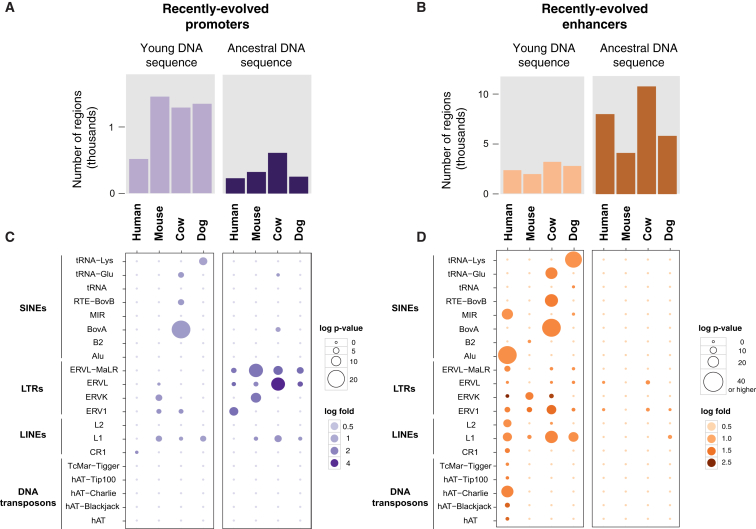
Recently Evolved Promoters Are Largely Derived from Young DNA, While Recently Evolved Enhancers Are Mostly Exapted from Ancestral DNA Sequences Regions with recently evolved promoter and enhancer activity in liver were identified in a representative species for each placental order (primate:human, rodent:mouse, ungulate:cow, and carnivore:dog). These regions were categorised into those falling in (1) young DNA sequences (0–40 Ma) or (2) ancestral DNA sequences (>100 Ma). (A) Typically three times as many recently evolved active promoters reside in young DNA as are found in ancestral DNA sequences present across placental mammals. (B) Conversely, typically twice as many recently evolved enhancers are exapted from evolutionarily ancestral DNA as are found in young DNA. (C and D) Repeat classes and families enriched in recently evolved promoters and enhancers were identified using a binomial test (see Experimental Procedures). Plots show enrichments for each repeat family (y axis) and each species (x axis). Circle sizes represent the statistical significance of enrichment, and color shades denote the fold change of the enrichment (both in logarithmic scale). See also Figures S6 and S7 and Tables S3, S4, S6, and S7.

From each placental order, we selected a representative species (human, mouse, cow, dog) and then identified a set of newly evolved or, more formally, apomorphic active promoters and enhancers in liver (Figure 6 and Figure S7). For each of these four species, we started with all active regions and then removed those that showed any activity within alignable regions in any other study species (see Experimental Procedures). We found that a typical mammalian liver deploys between 1,000 to 2,000 promoters and 10,000 enhancers not found in any other study species; we henceforth refer to these enhancers and promoters as *recently evolved*.

**Figure S6:**
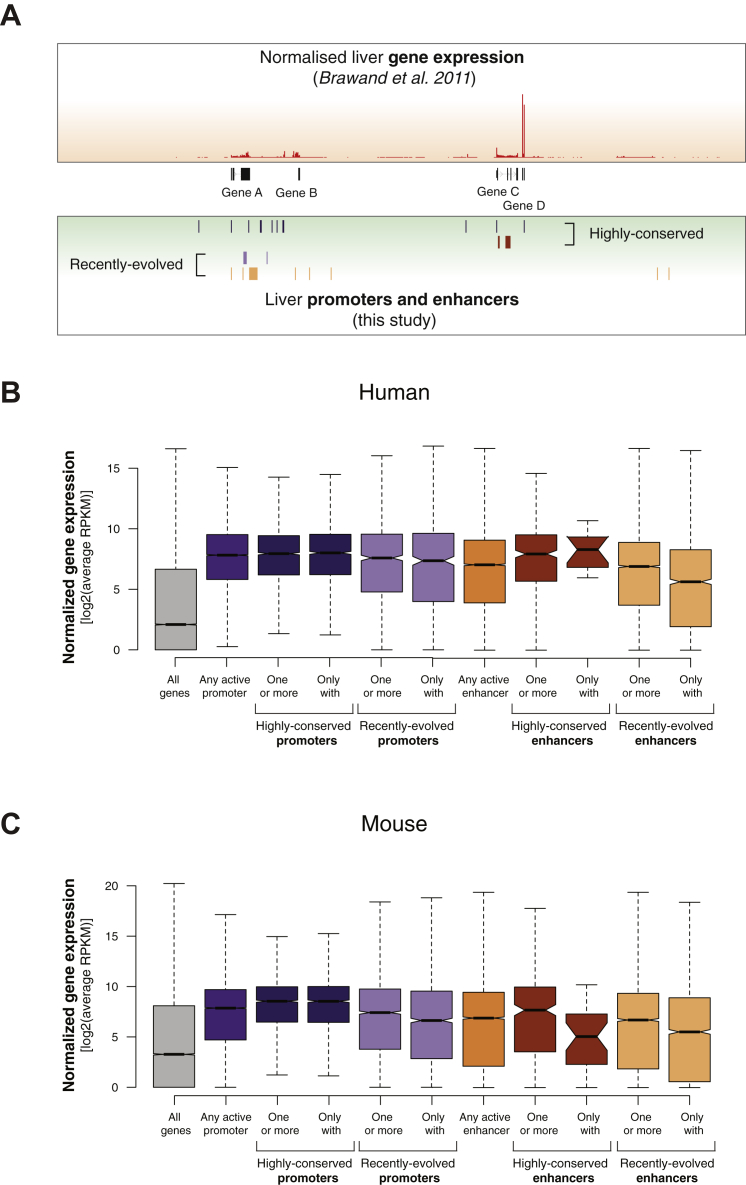
Expression Levels of Genes Associated to Highly Conserved or Recently Evolved Promoters and Enhancers, Related to Figures 5 and 6 (A) Previously reported gene expression data in human and mouse liver (Brawand et al., 2011) was integrated with highly conserved and recently evolved promoters and enhancers, as identified in this study using livers from the same species (see Experimental Procedures). (B and C) For human (B) and mouse (C), normalized gene expression levels (average RPKM, logarithmic scale) were quantified for genes associated with: (1) any promoter or enhancer active in liver, (2) at least one highly conserved promoter or enhancer, (3) only highly conserved promoter(s) or enhancer(s), or (4) and (5) the same associations with recently evolved promoters and enhancers. Liver promoter or enhancer activity is associated in all cases with gene expression levels above background (“All genes”).

These numbers are comparable to the extent of enhancer gains previously reported in inter-primate comparisons (Cotney et al., 2013; Shibata et al., 2012) and the extent of promoter evolution estimated from mouse-human comparisons (Forrest et al., 2014; Frith et al., 2006). Especially for enhancers, recently evolved regions are 10–20 times more abundant than those conserved across placentals or shared across multiple species in a particular lineage (Table S4). Both highly conserved and recently evolved regulatory regions active in liver are associated with increased expression of neighboring genes (Figure S6).

### Exaptation Drives Recently Evolved Enhancer, but Not Promoter, Activity

Using these tens of thousands of apomorphic regulatory regions, we tested whether functional exaptation of ancestral DNA, recently reported for human-specific enhancers active in embryonic limb (Cotney et al., 2013), is a prevalent mechanism in mammalian genome evolution.

**Figure S7:**
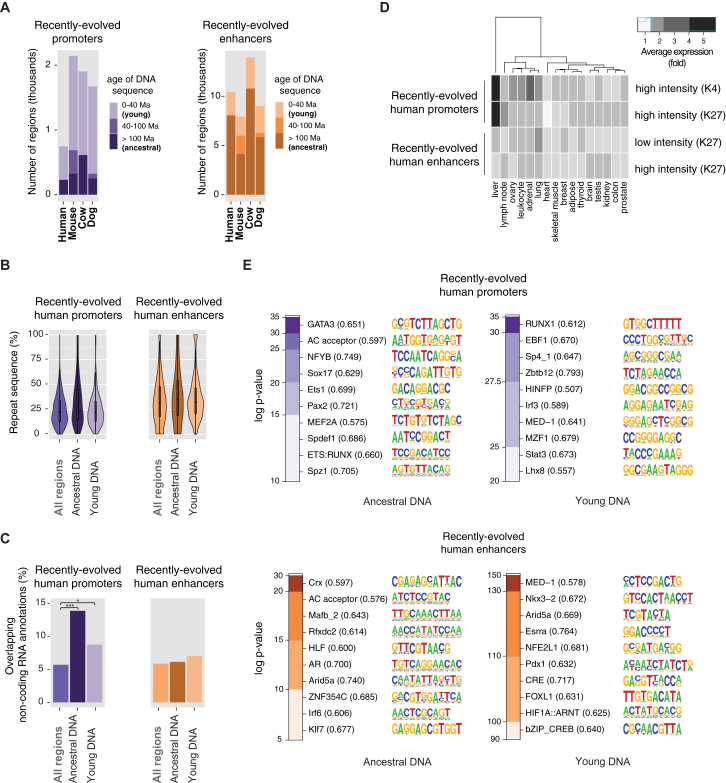
Additional Properties of Recently Evolved Promoters and Enhancers, Related to Figure 6 (A) Recently evolved promoters and enhancers identified in primates (human), rodents (mouse), ungulates (cow) and carnivores (dog) were categorised by the age of their underlying DNA sequence. Most recently evolved promoters and enhancers lie either in young DNA (0–40 Ma, lighter purple and orange shades) or ancestral DNA (> 100 Ma, darkest purple and orange), but a few promoters or enhancers lie in sequences of intermediate age (40–100 Ma). (B) Recently evolved promoters and enhancers contain similar proportions of sequences annotated as repetitive elements, regardless of the age of the underlying DNA, as shown for human in violin plots. For both promoters and enhancers, recently evolved elements located in ancestral or young DNA sequences were compared with all human promoters or enhancers (“All regions”). (C) Recently evolved promoters are significantly associated with non-coding RNA annotations, especially when lying in ancestral DNA sequences (p value < 0.0001, ancient DNA promoters; p value < 0.05, recent DNA promoters; proportion tests with Bonferroni correction). (D) Recently evolved human promoters associate with a high average expression in liver, compared to all identified promoters in human. Conversely, recently evolved human enhancers are not specifically enriched in liver-specific gene expression when compared to all enhancer elements identified in human (see also Figure S5 and Extended Experimental Procedures). Note that for simplicity low-intensity H3K4me3 and low-intensity H3K27ac promoters are not shown. (E) Sequence motifs enriched in recently evolved human promoters and enhancers residing in ancestral or young DNA, using all identified promoters or enhancers in human as a background control. Only the ten most enriched motifs are shown, and enrichment p values are represented as heatmaps (logarithmic scale).

We first asked whether recently evolved proximal promoters are primarily found in ancestral DNA sequences older than 100 Ma (Figure 6A, Figure S7). To our surprise, we discovered that across four orders of mammals, the recent evolution of promoters occurred within evolutionarily younger DNA segments (i.e., not shared with other study species) about three to four times as often as occurred by exaptation of ancestral DNA. For instance in mouse, 1,400 recently evolved promoters occurred in DNA sequences present only in this species (i.e., not shared even with rat); in contrast, only 260 recently evolved promoters were found in ancestral DNA.

Within the ancestral DNA commandeered into new promoters, and regardless of species interrogated, diverse ERV repeat elements are over-represented, consistent with previous reports that ERVs are pre-primed to transcriptional initiation (Fort et al., 2014).

In contrast, the vast majority of enhancers in liver are recently evolved (Table S4)—as well as far more likely to exapt ancestral DNA (Figure 6B). Of the typically 10,000 recently evolved enhancers in a given species, 52%–77% contained sequences of ancestral DNA over 100 Ma old. The remaining recently evolved enhancers were found in younger DNA, and enriched for mobile repetitive element families, including LTRs in all lineages and lineage-specific SINEs and DNA transposons exclusive to primates, carnivores, or ungulates (Figure 6B).

In a typical mammalian species, the 1,000 to 2,000 recently evolved liver promoters occur predominantly in younger DNA typically less than 40 Ma old, whereas the 10,000 recently evolved enhancers are formed predominantly by exaptation of ancestral DNA. Only a minority of recently evolved enhancers and promoters appear driven by repeat element expansions (Figure 6, Figure S7). Across our study's 20 mammals, exaptation of ancestral DNA generates more of the recently evolved regulatory genome than do repeat-driven expansions.

### Functional Annotation of Genes under Positive Selection

Comparing genome sequences can suggest which genes drive phenotypic adaptations by using inference of regions under positive selection and by analyzing amino acid substitution patterns in proteins (Nielsen et al., 2007). Both approaches primarily employ coding-sequence alignments and thus provide limited insight into regulatory adaptations. We therefore asked whether genes under positive selection are associated with apomorphic enhancers, perhaps evolving synergistically (Shibata et al., 2012).

We compared recently evolved enhancers and positively selected genes in two newly sequenced species: (1) naked mole rat, a cancer-resistant rodent (Kim et al., 2011); and (2) dolphin, a marine mammal metabolically adapted to an aquatic environment (Sun et al., 2013). In both species, we found that recently evolved enhancers are over-represented near positively selected genes (Experimental Procedures) (p values = 0.022 [naked mole rat] and 0.023 [dolphin], hypergeometric test. See Table S5).

**Figure 7:**
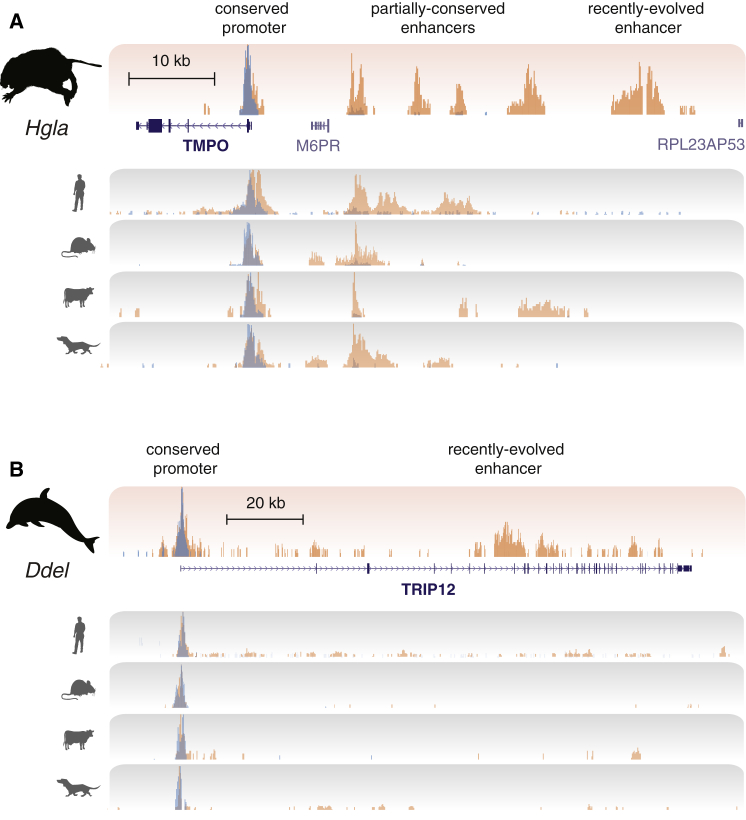
Recently Evolved Enhancers Associate with Genes under Positive Selection during Naked Mole Rat and Dolphin Evolution (A) The liver enhancer and promoter landscape surrounding the *TMPO* locus, which is under positive selection in naked mole rat (Kim et al., 2011), is shown (upper track). The bottom four tracks display overlaid H3K4me3 (blue) and H3K27ac (orange) levels in the orthologous regions of human, mouse, dog, and cow. Shown (left to right) are a promoter present in all species, four enhancer regions shared in a subset of species, and a naked mole rat-specific enhancer whose recently evolved activity is not present in other study species. (B) The enhancer and promoter landscape surrounding the *TRIP12* locus, which is under positive selection in dolphins (Sun et al., 2013), is shown. In this case, no mammals other than dolphin show liver enhancer activity near this gene; this enhancer is thus a good candidate to contain the regulatory regions associated with positive selection in dolphin. See also Table S5.

Illustrative examples are shown in Figure 7. First, a recently evolved enhancer in naked mole rat is shown upstream of the *thymopoietin* gene (*TMPO*), identified previously as positively selected (Kim et al., 2011). The orthologous *TMPO* regions in human, mouse, cow, and dog show no enhancer activity, though a number of partially conserved enhancers are present nearby (Figure 7A). Second, the genomic region around the *TRIP12* gene, under positive selection in dolphin (Sun et al., 2013), contains a recently evolved dolphin enhancer not active in human, mouse, dog, and cow. Moreover, this regulatory element appears to be the main enhancer in this region (Figure 7B).

In sum, recently evolved active regions identified in this study, and in particular rapidly evolving enhancers, can functionally annotate lineage-specific adaptations.

## Discussion

We experimentally dissected the evolution of regulatory regions in mammalian liver by mapping the genome-wide landscape of active promoters and enhancers from 20 diverse species. The evolutionary distances spanning four distinct orders within class *Mammalia* enabled rigorous analysis of the mechanisms underlying regulatory evolution. The combination of rapid enhancer and slower promoter evolution appears to be a fundamental property of the mammalian regulatory genome, shared by species separated by up to 180 million years. A sizable number of the 10,000–15,000 active promoters are functionally shared across most mammals, and are associated with ubiquitous cellular functions; highly conserved enhancers are much less common, and are found near liver-specific genes. Remarkably, almost half of 20,000–25,000 active enhancers in each species have rapidly evolved in a lineage- or species-specific manner. Our genome-wide mapping of enhancers in previously uncharacterized species has enabled us to identify regulatory regions near genes under positive selection that may help drive phenotypic adaptations.

### A Global Overview of Enhancer and Promoter Evolution in Mammals

We used a powerful and unbiased strategy to confirm, extend, and explicitly quantify previous results showing higher conservation of active promoter regions compared to distal enhancers in selected representatives of mammals (Xiao et al., 2012) or within primates (Cotney et al., 2013).

Our study has a number of limitations. First, the relationship between different histone marks and the activity of enhancers is not perfectly understood. Most active enhancers are marked by H3K27ac (Andersson et al., 2014; Creyghton et al., 2010; Zhu et al., 2013), and typically over two-thirds of regions enriched for H3K27ac show independent evidence in transgenesis assays for regulatory activity (Nord et al., 2013). Global mapping of H3K4me1 and p300 can also detect poised enhancer activity genome-wide, which can partly differ from that identified by H3K27ac (Heintzman et al., 2007; Krebs et al., 2011; Visel et al., 2009). Second, other approaches to map regulatory sequences, such as DNase-seq (Shibata et al., 2012) or ATAC-seq (Buenrostro et al., 2013), can reveal all regions of open chromatin genome-wide, but cannot distinguish promoters and enhancers. Third, our approach does not directly reveal which transcription factors control these regulatory regions, as would a more direct comparison (Kunarso et al., 2010; Paris et al., 2013; Schmidt et al., 2010), which in turn can only capture a modest subset of active regions. Fourth, our results generalize to other mammalian somatic tissues to the extent that adult liver is a representative tissue. However, other studies have suggested rapid enhancer evolution in mammals, using embryonic limb buds (Cotney et al., 2013), adipocytes (Mikkelsen et al., 2010), and embryonic stem cells (Xiao et al., 2012). These studies and others (Barbosa-Morais et al., 2012; Brawand et al., 2011) suggest that regulation in other somatic tissues evolves similarly, though embryonic tissues and their enhancers may be under stronger evolutionary constraint (Faure et al., 2012; He et al., 2011; Nord et al., 2013). Fifth, we cannot directly evaluate how often regions with regulatory activity are fully tissue-specific, particularly among those we assign as enhancers (Zhu et al., 2013).

One powerful strategy to dissect the regulatory genome has been to identify regions under high sequence constraint (Lindblad-Toh et al., 2011). Testing for activity has revealed that thousands of constrained noncoding regulatory sequences can act as enhancers in embryonic tissues (Pennacchio et al., 2006). The complementary approach we used additionally captures rapidly evolving regulatory regions. The enhancer regions we mapped likely range in function from essential to dispensible, which is reflected both in the modest sequence constraint and rapid evolution between species. Most of these regions would likely be missed by any sequence-conservation based approach. On the other hand, many DNA sequences we do not identify as enhancers may be active in other tissues or embryonic states, which we anticipate to be an area of active investigation.

Rapid enhancer and slow promoter evolution is a fundamental property of the mammalian regulatory genome. Active enhancer elements have a mean lifetime three times shorter than active promoters do, despite similar alignability of their underlying DNA sequences. Comparative sequence-based approaches have limited power to detect regulatory regions, in part because of their rapid evolution (Alföldi and Lindblad-Toh, 2013; Lindblad-Toh et al., 2011); indeed, our data indicate that sequence-based features such as sequence constraint or TF binding site density are poor predictors of enhancer conservation. Nevertheless, previous work across *Drosophila* species has indicated that specific TF motifs may be preferentially preserved in functionally conserved enhancers (Arnold et al., 2014). In agreement, we found motifs for the liver-specific transcription factor CEBPA enriched in highly conserved liver enhancers.

### Active Mammalian Enhancers Are Predominantly Apomorphic

Our results also newly reveal thousands of functionally active regulatory regions conserved across placental mammals, the vast majority of which are proximal promoter sequences. Placental-conserved proximal promoters in mammalian liver are commonly associated with ubiquitously expressed genes. In contrast, only 12% of highly conserved regulatory regions are active enhancers and these are near genes associated with liver-specific activities.

Perhaps our most surprising finding is that representative mammals typically deploy over 10,000 enhancers in a lineage- and probably most often species-specific manner. In total, almost half of all enhancers in each species appear to be recently evolved. Our results confirm and extend the concept that exaptation is a widespread phenomenon across placental mammals (Cotney et al., 2013), and redeployment of ancestral DNA is the dominant mechanism to generate active enhancers across a diverse cross-section of mammals. Interestingly, a recent study comparing enhancer activity across the much smaller genomes of five *Drosophila* species (Arnold et al., 2014) found a similar proportion of gained enhancers, especially for more distant species.

Another mechanism to create regulatory sequences is repeat-carried expansion of regulatory elements. Recent studies have indicated the involvement of specific repeat element expansions in the de novo creation of TF binding sites for CTCF (Bourque et al., 2008; Schmidt et al., 2012), Oct4/Nanog (Kunarso et al., 2010), and NRSF (Mortazavi et al., 2006). Our results show that repeat-carriage of newborn enhancers is not the dominant evolutionary process in mammals: repeat element enrichment is only significant among the recently evolved enhancers found in DNA less than 40 Ma old. Two technical limitations may have caused us to underestimate the repeat-driven creation of recently evolved enhancers (also, see Jacques et al., 2013): the difficulty of mapping reads to recently duplicated regions, and the incomplete representation of repeat regions in genome assemblies.

### Recently Evolved Promoters, Though Less Common Than Enhancers, Are Mostly Found in Young DNA

Promoters are far more evolutionarily stable than are enhancers. Nevertheless, the absolute number of promoters deeply conserved across all 20 study species is similar to the number of recently evolved promoters in any one species. Compared to the tens of thousands of newborn enhancers arising from exaptation of ancestral DNA, there are few newborn promoters—and these often arise from DNA sequences that are themselves evolutionarily young. We were not able to identify sequence features that account for the birth of promoters in young DNA. In contrast, the recently evolved promoters arising in ancestral sequences overlap LTR repeats, which enrich for latent non-coding RNA activity (Fort et al., 2014).

### A Strategy for Identifying the Enhancer Repertoire of Unannotated Genomes

Finally, extending an approach pioneered in well-annotated primate genomes (Cotney et al., 2013; Shibata et al., 2012), we provide examples of how experimental mapping of enhancers and promoters in newly sequenced mammals can annotate the regulatory network of genes, which have been identified computationally as under positive selection. Across representative species, we discovered that recently evolved enhancers are significantly over-represented in the vicinity of positively-selected genes and can often suggest candidate regulatory elements that could mediate species-specific adaptations. This result was obtained using only a single somatic tissue. Similarly, significant associations likely also exist in between the newly evolved enhancers specific to other somatic tissues and positively selected genes, which would uncover an extensive repertoire of highly evolvable, potentially synergistic regulatory connections.

### Future Directions

Our quantitation and analysis of the evolution of promoters and enhancers across a wide cross-section of mammals has revealed how dynamic and rapid enhancer evolution is. Within this regulatory diversity are the instructions by which a small number of founder species have radiated into surprising new niches, including marine (cetaceans) and aerial environments (bats). By combining detailed investigations of carefully selected sub-clades with new tools for modifying any sequenced genome, future studies will identify, formalize, and explore the functional instructions directing the diversity of mammalian forms.

## Experimental Procedures

We performed ChIP-seq using liver tissue isolated from 20 mammalian species (Table S1). At least two independent biological replicates from different animals, generally young adult males, were performed for each species and antibody. The only exception was *Balaenoptera borealis*, for which a single individual was profiled, and dolphin, for which we profiled a single individual from two closely-related species. ChIP-seq experiments were performed as recently described (Aldridge et al., 2013) with antibodies against H3K4me3 (Millipore 05-1339) and H3K27ac (Abcam ab4729). To match inter-individual variability for the two histone marks, the same tissue samples were used for both antibodies and control input DNA in each species.

Sequencing reads were aligned to the appropriate reference genome with BWA v.0.5.9 (Table S2) and regions of enrichment determined with MACS v1.4.2. Regions enriched in two to four biological replicates and overlapping by a minimum 50% of their length were merged and categorized into active promoters (H3K4me3-enriched regions, with or without overlapping H3K27ac enrichment) or enhancers (regions enriched only for H3K27ac). Cross-species comparisons were performed through the Ensembl API. Human, macaque, vervet, marmoset, mouse, rat, rabbit, cow, pig, dog, and cat were directly cross-compared using the 13 eutherian mammals EPO alignment available from Ensembl (Flicek et al., 2014). Species not included in the EPO alignment were compared to the reference species of their respective clade (human, mouse, cow, dog, or opossum) using Lastz aligments. Promoters or enhancers were considered as having conserved activity between species when their orthologous location in the second species overlapped a marked region by a minimum of 50% in length. All pairwise comparisons correspond to average values of reciprocal comparisons between species. Genome annotations (including gene ontology and repetitive and constrained elements) were downloaded from Ensembl v73. See also Extended Experimental Procedures.

Extended Experimental Procedures All scripts used for computational analyses were written in Perl (http://www.perl.org), Python (http://www.python.org), R (http://www.r-project.org; Team, 2011), or Bioconductor (http://www.bioconductor.org; Gentleman et al., 2004), using Ensembl API packages and R packages GenomicRanges, ShortRead, Sgenome, Biostrings, gtools, gplots, extraLattice, scales, vioplot, plotrix, limma, ape, geiger, reshape2 and ggplot2.

**Source and Detail of Tissues**

We performed chromatin immunoprecipitation experiments followed by high throughput sequencing (ChIP-seq) using liver tissue isolated from 20 mammalian species. The origin, number of replicates, sex, and age for each species’ samples are detailed in Table S1.

At least two independent biological replicates from different animals were performed for each species and antibody. The only exception was *Balaenoptera borealis*, for which a single individual was profiled. For the two closely-related dolphin species *Delphinus delphis* and *Lagenorhynchus albirostris*, we profiled one individual of each species and treated them as two dolphin biological replicates.

Wherever possible, livers from young adult males were used. Tissues from ten species were excess from routine euthanasia procedures (e.g., from individuals sacrificed during maintenance of research colonies). Five species were purchased commercially (for instance, from slaughterhouses). Specialty conservation programmes (e.g., zoos and cetacean stranding post-mortems) often collect tissues for research purposes, and we obtained four species’ tissues from these efforts. Samples of healthy liver tissue from humans were obtained from the Addenbrooke’s Hospital at the University of Cambridge under license number 08-H0308-117 ‘‘Liver specific transcriptional regulation’’. Mouse samples were obtained from the Cambridge Institute under Home Office license PPL 80/2197. With the exception of the *Lagenorhynchus albirostris* sample, cetacean tissues were from stranded individuals that died on the beach and were in a freshly dead condition at the time of post-mortem.

In almost all cases, tissues were prepared immediately post-mortem (typically within an hour) to maximize experimental quality. Post-mortem tissues were kept on ice until processed to minimize potential loss of protein-DNA interactions during post-mortem time.

**Chromatin Immunoprecipitation and High-Throughput Sequencing**

For fresh tissue samples (see Table S1), hepatocytes were prepared by direct perfusion of the liver with PBS, followed by cross-linking of the diced tissue in 1% formaldehyde solution for 20 min, addition of 250 mM glycine and incubation for a further 10 min to neutralize the formaldehyde. Liver samples from frozen specimens were powdered while frozen by using a mortar and pestle on dry-ice, and the powdered frozen tissue was subsequently cross-linked as described above. After homogenization of cross-linked liver tissue in a dounce tissue grinder, hepatocytes were rinsed with PBS twice and lysed according to published protocols (Schmidt et al., 2009) to solubilize DNA-protein complexes. Chromatin was fragmented to 300 bp average size by sonication on a Misonix sonicator 3000 with a 418 tip. Chromatin from 0.1 g of dounced liver tissue was used for each ChIP experiment using antibodies against H3K4me3 (millipore 05-1339) and H3K27ac (abcam ab4729) in an Agilent Bravo liquid handling robot (Aldridge et al., 2013). Illumina sequencing libraries were prepared from ChIP-enriched DNA in 96 well microtiter plates using automated liquid handling robotic platforms (Quail et al., 2008). 10 PCR cycles were used for input DNA (500 ng) and 15 cycles for ChIP DNA. After PCR, libraries were pooled in equimolar concentrations and sequenced on an Illumina HiSeq 2000 for 50 cycles single end, plus index read.

**Short-Read Alignment and Peak Calling**

Sequencing reads were aligned to the appropriate reference genome (see Table S1) using BWA v.0.5.9 with default parameters (Li and Durbin, 2009). Low-quality and multiple-mapping reads were removed using Samtools with option “-q 1” (Li et al., 2009). Aligned read counts were normalized to 10 million uniquely mapped reads per experiment, by subsampling of the alignment files. Enriched regions (or peaks) were called using MACS v.1.4.2 with default parameters (Zhang et al., 2008), using total DNA input as control and retaining all statistically enriched regions (p < 10^−5^; no filtering on fold enrichment or FDR correction). Enriched regions were considered as reproducible when they were identified in at least two biological replicates and overlapped by a minimum 50% of their length. Consensus peaks were then built by merging these overlapping regions across all replicates. Non-reproducible regions were discarded for the main analyses (except for *Balaenoptera borealis*, for which only one biological replicate was available and in which all enriched regions were retained). Peak intensity values in Figure S1 were calculated as the mean fold enrichment reported by MACS across replicates.

H3K4me3 and H3K27ac consensus peaks in each species were overlapped to determine genomic regions enriched for H3K4me3, H3K27ac or both. Double-marked H3K4me3&H3K27ac elements were identified as regions reproducibly marked by H3K4me3 and H3K27ac and overlapping by a minimum 50% of their length, and were merged as above.

**Cross-Species Comparisons**

Pairwise comparisons were performed by mapping enriched ChIP-seq regions between species in a reciprocal manner using whole-genome alignments. Human, macaque (and vervet), marmoset, mouse, rat, rabbit, cow, pig, dog, and cat were cross-compared using the 13 eutherian mammals EPO alignment available from Ensembl (Paten et al., 2008). Additional species not included in the EPO alignment were compared to both human and the reference species of their respective clade (human, mouse, cow, dog or opossum) using Lastz alignments, in a strategy similar to the building of the EPO_LOW_COVERAGE alignment available from Ensembl (Flicek et al., 2014). All comparisons were performed through the Ensembl API using custom Perl scripts.

Regions that could not be unambiguously mapped to orthologous locations in the other genome (i.e., regions split over multiple alignment blocks) were discarded from the comparison. Marked regions were considered as functionally conserved between species when their orthologous location in the second species overlapped a marked region by a minimum of 50%. Of note, the minimum overlap used had little influence over the number of conserved regions obtained, and minimum required overlaps ranging from 1 to 80% gave very similar results to those reported here. All pairwise comparison values correspond to the average of reciprocal comparisons between both species (e.g., human peaks conserved in dog and dog peaks conserved in human).

**Human-Centric Inter-Species Analysis**

For each promoter (H3K4me3&H3K27ac or H3K4me3 only) or enhancer (H3K27ac only) experimentally identified in human liver, the number of species in which an orthologous sequence exists was determined using either the EPO multiple alignments (for ten species) or LastZ alignments of all other species with human (Figure 2). This measure used only the human ChIP-seq data and provides a maximum threshold for the functional conservation of each human regulatory region, based on the alignability of its DNA to the genomes of the other 19 species. Then the number of species in which a human promoter or enhancer is functionally conserved was measured by comparing the human peak with the ChIP-seq signal in the orthologous locations from all other species; this measure used ChIP-seq data from all 20 species. Conservation of promoter or enhancer activity was then evaluated by comparing the number of species in which the region was functionally conserved (as described above) to the number of species in which its DNA sequence was alignable. Naked mole rat alignments with human were not available in Ensembl, and for this species we mapped functional conservation by projecting the data to human using the liftOver tool from UCSC, with a 50% minimum overlap.

**Multiple Regression Analysis**

The conservation ratio of each human promoter or enhancer was determined as the number of species with conserved activity divided by the number of mapped species (see above and Figure 3). These conservation values were modeled as a function of experimental and genomic properties of each promoter or enhancer using multiple linear regression analysis. Experimental reproducibility was the fraction of replicates where an enriched region was found, and peak intensity was calculated as in Figure S1. Sequence constraint was estimated as the percentage of bases having rejected substitutions (according to GERP, (Cooper et al., 2005)), and predicted transcription factor binding sites were obtained with FIMO software (Grant et al., 2011), using the Transfac 10.2 motif database (q-value ≤ 0.1). The inter-dependences among these properties was evaluated by Pearson correlation.

**Empirically Determined Rates of Divergence**

Pairwise conservation ratios of promoters, enhancers and CEBPA binding sites were calculated from pairwise comparisons between species, and the average value of the two reciprocal comparisons is reported in Figures 4 and Figure S4. Conservation ratios were plotted along divergence times between species, according to the mammalian phylogeny in Ensembl v73. Half lives and mean life times for each class of regulatory element were estimated from an exponential decay fit. For promoters and enhancers, we used (1) data from the ten species with highest genome qualities in the 13 eutherian mammals EPO multiple alignment (Figure 4) or (2) from all 20 species (Figure S4) using a combination of EPO and LastZ pairwise alignments (see above). For CEBPA, we used previously reported data in five mammals (Schmidt et al., 2010). Rates of divergence values in Figure 4B were almost identical when data from all species was used (Figure S4). Neighbor-Joining trees were built based on pairwise distance matrices corresponding to the proportion of non-conserved promoters or enhancers between pairs of species using the ape R library.

**Identification of Highly Conserved Regions**

Regulatory regions functionally conserved across placental mammals were defined as orthologous regions showing ChIP-seq enrichment across all ten species in the Ensembl EPO multiple alignment (Figure 5). Human was used as an anchor species: each human promoter and enhancer was tested for marking across the 19 other species (see above), and identified as a “highly conserved element” when orthologous regions were consistently enriched with either or both histone marks in all ten highest-quality genomes, plus any other additional species. Highly conserved elements were then assigned as “highly conserved promoters” or “highly conserved enhancers” by a majority rule, depending on the histone mark(s) most often observed across species (H3K4me3 and H3K27ac for promoters, and H3K27ac only for enhancers).

The number of identified highly conserved elements were compared to random expectation by a permutation test with 10,000 iterations (random permutations of the regions conserved with human for each species and each histone mark independently), counting the number of randomly expected promoters and enhancers conserved across at least all ten high-quality genomes. Sequence constraint in each highly conserved region was determined as the percentage of human bases identified by GERP (Cooper et al., 2005) as having rejected substitutions.

**Identification of Lineage- and Recently Evolved Regions**

Lineage-specific conservation of regulatory regions (Table S4) was determined for primates, rodents, ungulates, and carnivores using a similar strategy as that for highly conserved elements (Figure 6). ChIP-seq enriched regions were compared between a reference species (human, mouse, cow, and dog) and other species in the clade using either the EPO multiple alignment when possible or pairwise Lastz alignments otherwise. Elements functionally conserved across the high-quality genomes in each lineage, but not in any other species, were identified for each histone mark (i.e., in human, macaque, and marmoset for primates; mouse, rat, and rabbit for rodents; cow and pig for ungulates; and dog and cat for carnivores). These were then categorised into lineage-specific promoters and enhancers based on their dominant histone mark enrichment across species within the clade, as described above.

Recently evolved promoters and enhancers were determined for a reference species in each lineage (human, mouse, cow, and dog). Enriched regions in the reference species that showed functional conservation in any alignable species were discarded. The number of species that were used for comparison with each reference species was 18 (human), 12 (mouse), 12 (cow) and 10 (dog). These include: (1) nine species in the 13 eutherian mammals EPO multiple alignment, (2) other species within the clade, evaluated with ad hoc LastZ pairwise alignments with the reference species (e.g., mouse-guinea pig, mouse-naked mole rat and mouse-tree shrew) and (3) all other species but naked mole rat for human, using pairwise LastZ alignments. Recently evolved elements were then categorised into promoters and enhancers by overlapping the two histone marks in each reference species.

Recently evolved elements were similarly identified for two non-reference species (naked mole rat and dolphin). When the number of genomic alignments available for a species was small (e.g., for dolphin, only alignments with human and cow were available), we additionally mapped the promoters and enhancers of the species of interest to their orthologous locations in the reference species of its clade (in this case, cow) and tested whether they correspond to marked regions in any other species in the EPO alignment.

**Sequence Age and Repeat Enrichment Analysis**

Sequence age analysis of recently evolved promoters and enhancers was adapted from the approach reported by (Cotney et al., 2013) (Figures 2 and S7). Briefly, the sequence age of a recently evolved element was estimated from the most distantly related species with an alignable orthologous sequence, using cross-species comparisons as described above. These alignments allowed categorisation of ages into recently evolved DNA (0–40 Ma, ranging from recently evolved sequence to sequences shared with the closest species in the dataset), 40–100 Ma DNA (within the evolutionary distances found in each lineage) or ancient DNA (≥100 Ma, and thus as old or older than the placental radiation). For clarity, only the first and last are reported in Figure 6, with all three being shown in Figure S7.

Repetitive element families over-represented in recently evolved promoters and enhancers were evaluated using RepeatMasker annotations (Smit et al., 1996–2010) in each reference species, obtained from the UCSC Table Browser for assemblies GRCh37/hg19, GRCm38/mm10, UMD3.1/bosTau6 and CanFam3.1/canFam3. Enrichment of specific repetitive element families was assessed using a binomial test with Benjamini-Hochberg FDR correction for multiple testing, with all experimentally defined promoters or enhancers in each reference species used as expected background. Repetitive elements were considered as included in a promoter or enhancer if they overlapped by a minimum of 50%.

**Gene Annotation, Gene Ontology Analysis**

Gene annotations were downloaded from Ensembl v73 (Flicek et al., 2014) and associated with regions of ChIP enrichment using the default association rule proposed in GREAT (McLean et al., 2010), in which gene regulatory domains extend in both directions to the proximal promoter of the nearest gene (−5 kb/+1 kb from the transcription start site ie. TSS), but no more than 1 Mb in either direction (Figures 5, 6, 7, S5, S6, S7, and Tables S3 and S6). A single consensus transcript (and therefore TSS) annotation was used for each gene, as defined by Ensembl. Gene domains associated to ChIP-defined promoters and enhancers were then used for gene ontology analysis (Figure S5 and Table S6), association with liver-specific genes (related to Figures 5 and 6) or genes under positive selection (Figure 7).

Gene ontology analysis was performed using gene ontology annotations from Ensembl v73. Enrichment of ontology annotations around specific categories of enhancers or promoters was evaluated using a binomial test with Benjamini-Hochberg FDR correction for multiple testing. Only terms with corrected p values lower than 0.05 and fold enrichments greater than two are reported. This method is similar to the gene ontology analysis available in GREAT for human and mouse.

For Figure 5B, the distance of highly conserved elements to the nearest TSS was determined using human gene annotations in the GRCh37.p12/hg19 Ensembl assembly, including both coding and non-coding annotations but filtering out pseudo and introgressed genes. Of note, only the TSS of the consensus gene annotations available from Ensembl were used; potential alternative TSSs were not included. A similar approach was used in Figure S2 for experimentally defined promoters and enhancers in each species. Non-coding RNA annotations in Figure S7 were selected using BioMart, and included all non-coding RNA categories (long, miRNA, etc).

**Regulatory Annotation of Genes under Positive Selection**

Enrichment of previously reported positively selected genes (PSGs) in the vicinity of recently evolved enhancers was assessed using both hypergeometric and proportion tests (Figure 7 and Table S5). The number of PSGs identified in each species was small (typically less than 100), and reported p values were not corrected for multiple testing. We performed several tests to evaluate the robustness of observed enrichments. Hypergeometric tests were performed in both directions, to evaluate (1) whether recently evolved enhancers are significantly more likely to occur in the regulatory domains of PSGs in each species and (2) whether recently evolved PSGs are more likely to harbor (at least one) recently evolved enhancer(s) than other genes. We additionally used Wilcoxon’s test to ask whether the regulatory domains of PSGs contain a higher average proportion of recently evolved enhancers, compared to those of non-PSGs.

**Gene Expression Analysis**

Enrichment of liver-specific genes in the proximity of highly conserved or human-specific promoters and enhancers was evaluated as above, using a combination of hypergeometric and Wilcoxon’s tests (Figures S5, S6, and S7 and Table S3). We identified a set of liver-specific genes from previously published RNA-seq data across human tissues (Petryszak et al., 2014), using a similar strategy as in (Cotney et al., 2013; McLean et al., 2010). For all represented human genes, we calculated tissue-specificity scores (tsps) as previously described (Ravasi et al., 2010). We then selected liver-specific genes as those having (1) a tsps above 1.5, (2) its highest expression in liver and (3) an RPKM value above 10 in liver.

For Figures S5 and S7, we also calculated the average expression of genes associated with highly conserved or human-specific promoters and enhancers, as a ratio over that found in all human promoters/enhancers. For the calculation of average expression values, genes having no expression measurements in the RNA-seq data for a particular tissue were assumed to be not expressed (RPKM = 0).

Normalized gene expression levels in human and mouse liver were retrieved from (Brawand et al., 2011). For Figure S6, we compared the expression of sets of genes based on the conservation of their associated promoters and enhancers, as described above. The expression value for each gene was calculated as the average RPKM value over the two or three replicates in the original study.

**Motif Enrichment Analysis**

Short sequence motifs enriched in highly conserved and recently evolved promoters/enhancers were indentified with Homer (Heinz et al., 2010) (Figures S5 and S7 and Table S7). Briefly, enriched motifs were identified de novo and compared with known transcription factor binding site profiles (Portales-Casamar et al., 2010). We used either random GC- and length-matched sequences or all promoters or enhancers identified in the same species as the background set; thus testing for motif enrichments (1) compared to random expectation and (2) specific to highly conserved or recently evolved elements.

## Author Contributions

D.V., C.B., P.F., and D.T.O. designed experiments; D.V. and S.A. performed experiments; C.B., D.V., T.F.R.,and M.L. analyzed the data; T.J.P., R.D., J.T.E., A.J.J., J.M.A.T., M.F.B., and E.P.M. provided tissue samples; M.P. generated LastZ whole-genome alignments; D.V., C.B., P.F., and D.T.O. wrote the manuscript; P.F. and D.T.O. oversaw the work. All authors read and approved the final manuscript.

## Accession Numbers

Data have been deposited under ArrayExpress accession number E-MTAB-2633.

## Supplemental Information

Document S1. Tables S1–S5

Table S6. Gene Ontology Enrichment Analyses, Related to Figures 5, 6, S7, and S9

Table S7. Sequence Motifs Enrichment Analyses, Related to Figures 5, 6, S7, and S9

Document S2. Article plus Supplemental Information
