# Supplementary material for: Enhancer Evolution across 20 Mammalian Species
Source: Cell. 2015 Jan 29;160(3):554–66. doi: 10.1016/j.cell.2015.01.006 (PMC4313353; doi:10.1016/j.cell.2015.01.006)
Supplement: Document S1. Tables S1–S5 [file mmc1.pdf]

Table S1. Species and Tissue Samples Used in This Study, Related to Figure 1 and Experimental Procedures

**Species common name**[a]	**Species scientific name**	**Age of sexual maturity / lifespan**	**Provider**	**Provider class**	**Number of replicates**	**Sex**	**Age**	**Age group**	**Tissue preparation**[b]
Human **Hsap**	*Homo sapiens*	12-15 years / 80 years	Addenbrookes Hospital (UK)	Hospital	4	All M	unknown	adult	fresh
Macaque **Mmul**	*Macaca mulatta*	4 years / 20 years	Medical Research Council (UK)	Research colony	4	M, M, M, F	unknown, 18, 5, 11 (years)	adult, juvenile	fresh
Vervet **Csab**	*Chlorocebus aethiops sabaeus*	2-5 years / 11-13 years	Vervet Research Colony (US)	Research colony	3	All F	8, 9, 9 (years)	adult	fresh
Marmoset **Cjac**	*Callithrix jacchus*	1.5 years / 12 years	Harlan ltd (UK)	Research colony	3	All M	unknown	adult	fresh
Mouse **Mmus**	*Mus musculus domesticus*	6-8 weeks / 1-3 years	Charles river (UK)	Research colony	4	All M	10 weeks	adult	fresh
Rat **Rnor**	*Rattus norvegicus*	5 weeks / 1-3 years	Harlan ltd (UK)	Research colony	3	All M	10 weeks	adult	fresh
Guinea pig **Cpor**	*Cavia porcellus*	3-5 weeks / 4-8 years	Harlan ltd (UK)	Research colony	3	All M	10 weeks	adult	fresh
Naked mole rat **Hgla**	*Heterocephalus glaber*	8-12 months / 30 years	UIC (US)	Research colony	3	All M	1 year	adult	fresh
Rabbit **Ocun**	*Oryctolagus cuniculus*	5-6 months / 8-12 years	Harlan ltd (UK)	Research colony	3	All M	7,12,12 (months)	juvenile, adults	fresh
Tree shrew **Tbel**	*Tupaia belangeri*	4-5 months / 9-12 years	Cardiff University (UK)	Research colony	3	M, M, F	16, 3, 6 (months)	adult, juveniles	fresh
Cow **Btau**	*Bos taurus*	8-12 months / 15 years	B&K ltd (UK)	Commercia l	4	All M	2, 1.5, 2, 2 (years)	adult	fresh

Species common name[a]	Species scientific name	Age of sexual maturity / lifespan	Provider	Provider class	Number of replicates	Sex	Age	Age group	Tissue preparation[b]
Dolphin **Ddel** (short-beaked common dolphin and white-beaked dolphin)	*Delphinus delphis*	12-15 years / 22 years	UK Cetacean Strandings Investigation Programme, Zoological Society of London (UK)	Specialised research programme	1	M	unknown	adult	frozen
	Lagenorhynchus albirostris	uknown / 25 years			1	F	unknown	adult	frozen
Sei whale **Bbor**	*Balaenoptera borealis*	8-10 years / 50-70 years			1	F	unknown	juvenile	frozen
Sowerby's beaked whale **Mbid**	*Mesoplodon bidens*	7 years / unknown			2	Both F	unknown	juvenile	frozen
Pig **Sscr**	*Sus scrofa*	6 months / 10-15 years	Harlan ltd (UK)	Research colony	3	All M	2 years	adult	fresh
Dog **Cfam**	*Canis familiaris*	1 year / 12-15 years	Harlan ltd (UK)	Research colony	3	All M	2.5, 1, 1 (years)	adult, juveniles	fresh
Cat **Fcat**	*Felis catus*	5-10 months / 15 years	Isoquimen ltd (Spain)	Research colony	2	Both F	1.5 years	adult	fresh
Ferret **Mfur**	*Mustela putorius furo*	6 months / 8 years	B&K ltd (UK)	Research colony	3	All M	8, 6, 6 (months)	adult, juveniles	fresh
Opossum **Mdom**	*Monodelphis domestica*	4-5 months / 4-8 years	MRC National Institute for Medical Research (UK)	Research colony	3	All M	6 months	juveniles	fresh
Tasmanian Devil **Shar**	*Sarcophilus harrisii*	2 years / 5-6 years	Copenhagen Zoo (Denmark)	Zoo	2	F, M	8, 7.5 (years)	adult	frozen

[a] Species abbreviations used in the manuscript are given in bold
[b] see Extended Experimental Procedures (Chromatin immunoprecipitation and high through-put sequencing).

Table S2. Quality Assessment of Reads Alignments and Peaks Detection, Related to Figure 1 and Experimental Procedures

Species	Alignment genome and version[a]	Control reads[b]		Peaks reproducibility (%)[c]		Replicate-specific peaks[d]	
		Aligned (%)	Uniquely aligned (%)	H3K4me3	H3K27ac	H3K4me3	H3K27ac
Human	GRCh37.p12/hg19	95.1	84.1	94.7	77.0	660	11,533
Macaque	MMUL1.0/rheMac2	96.8	78.4	87.6	73.9	1,535	10,183
Vervet	MMUL1.0/rheMac2	84.5	64.4	65.5	73.4	6,093	9,341
Marmoset	C_jacchus3.2.1/calJac3	96.8	84.8	80.5	78.5	3,549	11,258
Mouse	GRCm38.p2/mm10	98.7	73.3	96.5	85.7	524	4,656
Rat	Rnor5.0/rn5	96.9	76.2	79.4	80.5	4,552	8,350
Guinea pig	cavPor3	89.0	68.0	95.6	83.8	584	6,775
Naked mole rat	HetGla1.0/hetGla2	98.2	85.0	93.2	84.6	969	6,301
Rabbit	OryCun2.0/oryCun2	92.7	63.7	68.9	63.7	4,065	14,628
Tree shrew	tupBel1	77.8	54.2	89.0	82.9	1,904	6,445
Cow	UMD3.1/bosTau6	98.4	73.8	94.2	74.0	786	13,645
Dolphin	turTru1	90.5	67.0	74.8	63.8	6,646	17,022
Sei whale	turTru1	61.4	37.6	-	-	-	-
Beaked whale	turTru1	62.0	34.1	62.4	69.8	8,769	12,377
Pig	Sscrofa10.2/susScr3	88.3	69.7	87.7	77.0	1,515	9,380
Dog	CanFam3.1/canFam3	97.5	89.1	93.9	83.6	716	5,608
Cat	Felis_catus_6.2/felCat5	96.3	89.2	89.2	78.1	1,308	8,849
Ferret	MusPutFur1.0/musFur1	91.6	84.8	80.4	77.2	3,973	9,420
Opossum	monDom5	96.2	82.8	88.1	79.0	2,208	9,717
Tasmanian Devil	Devil_7.0/sarHar1	96.2	83.1	84.6	67.5	2,523	14,674

[a] Ensembl genome versions were used, and the equivalent UCSC genome version is given after a slash. Both macaque and vervet ChIP-seq reads were aligned to the *Macaca mulatta* macaque genome, and all reads from all cetacean samples were aligned to the *Tursiops truncatus* dolphin genome.

[b] Average percentage of reads in the control experiments (total DNA) that can be either aligned or uniquely aligned to the reference genome.

[c] Average percentage of peaks per experiment that are replicated in at least one other experiment (see Experimental Procedures, Short reads alignment and peak calling).

[d] Average number of peaks specific to an individual, i.e. not replicated in any other experiment.

Table S3: **Association Tests between Liver-Specific Genes and Highly-Conserved or Recently-Evolved Human Promoters and Enhancers, Related to Figures 5 and 6**

	**Wilcoxon test** Mean proportion of promoters/enhancers in:			**Hypergeometric test** Promoters/enhancers associated with liver-specific genes					**Hypergeometric test** Liver-specific genes associated with promoters/enhancers				
**Genomic elements set**	**Liver-specific**[a]	**All genes**	**p-value**	**Elements in liver-sp genes**	**Total elements**	**All other elements**	**Total elements in liver-sp genes**	**p-value**	**Liver-sp genes within**	**All liver-sp genes**	**All other genes**	**Genes within**	**p-value**
Highly-conserved promoters	0.011	0.044	0.7965	53	1871	9732	313	0.3140	47	228	9,153	1,908	0.4196
Highly-conserved enhancers	0.027	0.009	**0.0097**	16	279	28,813	968	**0.0127**	16	215	7,084	294	**0.0059**
Recently-evolved human promoters	0.059	0.037	**0.0325**	34	783	11,013	316	**0.0019**	33	228	9,153	778	**0.0005**
Recently-evolved human enhancers	0.414	0.505	0.6956	274	10,363	18,729	968	0.9999	124	215	7,084	4,839	0.9954

[a] Liver-specific genes were identified as described in Methods, using publicly available expression data from 16 human tissues (Petryszak et al., 2014).

Table S4. Number of Lineage-Specific and Recently-Evolved Elements Identified in Primates, Rodents, Ungulates and Carnivores, Related to Figure 6

	STRICT ASSESSMENT OF ACTIVITY CONSERVATION (REPRODUCIBLE ORTHOLOGOUS PEAKS)[a]								
	Species[b]	Promoters[c]	Highly-conserved	Lineage-specific	Recently-evolved	Enhancers[c]	Highly-conserved	Lineage-specific	Recently-evolved
PRIMATES	**Human**	11,613	1,872	13	794	29,177	279	352	10,434
	Macaque	10,729				22,911			
	Vervet	9,704				16,769			
	Marmoset	10,808				31,649			
RODENTS	**Mouse**	12,443		121	2,847	18,561		136	7,930
	Rat	13,782				22,471			
	Guinea pig	11,986				25,153			
	Naked mole rat	11,300				25,238			
	Rabbit	8,790				19,115			
	Tree shrew	13,426				19,642			
UNGULATES	**Cow**	12,972		275	2,108	32,371		928	13,929
	Dolphin	15,019				14,770			
	Sei whale	17,045				34,381			
	Beaked whale	11,653				16,432			
	Pig	10,103				23,822			
CARNIVORES	**Dog**	10,329		165	1,793	20,220		734	9,012
	Cat	9,355				22,238			
	Ferret	13,140				21,377			
MARSUPIALS	Opossum	15,211				23,271			
	Tasmanian devil	10,496				19,983			

	LENIENT ASSESSMENT OF ACTIVITY CONSERVATION (ANY SIGNAL AT ANY ORTHOLOGOUS LOCATION)[a]								
	Species[b]	Promoters[c]	Highly-conserved	Lineage-specific	Recently-evolved	Enhancers[c]	Highly-conserved	Lineage-specific	Recently-evolved
PRIMATES	**Human**	11,613	2,457	10	737	29,177	684	356	8,699
	Macaque	10,729				22,911			
	Vervet	9,704				16,769			
	Marmoset	10,808				31,649			
RODENTS	**Mouse**	12,443		131	2,602	18,561		202	6,891
	Rat	13,782				22,471			
	Guinea pig	11,986				25,153			
	Naked mole rat	11,300				25,238			
	Rabbit	8,790				19,115			
	Tree shrew	13,426				19,642			
UNGULATES	**Cow**	12,972		147	1,941	32,371		1021	12,111
	Dolphin	15,019				14,770			
	Sei whale	17,045				34,381			
	Beaked whale	11,653				16,432			
	Pig	10,103				23,822			
CARNIVORES	**Dog**	10,329		37	1,695	20,220		736	7,818
	Cat	9,355				22,238			
	Ferret	13,140				21,377			
MARSUPIALS	Opossum	15,211				23,271			
	Tasmanian devil	10,496				19,983			

[a] Strict assessment: an active region in species A is defined as conserved in species B if the orthologous locus in species B was reproducibly active in two replicates or more. Lenient assessment: an active region in species A is defined as conserved in species B if any replicate in species B showed significant activity at the orthologous locus ($p < 10^{-5}$, no FDR correction).

[b] Reference species in each lineage are indicated in bold. Additional species in the EPO multiple alignment, used to identify highly-conserved elements, are indicated in blue.

[c] Numbers given are total promoters or enhancers experimentally identified from each species' ChIP-seq data.

Table S5. Association Tests between Positively Selected Genes (PSGs) and Recently-Evolved Enhancers in Naked Mole Rat and Dolphin, Related to Figure 7

		**Wilcoxon test** Mean proportion of recently-evolved enhancers in:			**Hypergeometric test** Recently-evolved enhancers associated with PSGs					**Hypergeometric test** PSGs associated with recently-evolved enhancers				
**PSG set**	**Recently-evolved enhancers**	**PSGs**	**All genes**	**p-value**	**Rec-ev in PSGs**	**Rec-ev enhancers**	**All other enhancers**	**Total enhancers in PSGs**	**p-value**	**PSGs with rec-ev enhancers**	**PSGs**	**All other genes**	**Genes with rec-ev enhancers**	**p-value**
Dolphin_Sun[a]	Dolphin	0.0643	0.0360	**0.059**	9	614	1580	24	0.104	8	101	21225	878	**0.023**
Whale_Yim[b]	Dolphin	0.0386	0.0360	0.651	13	614	1580	39	0.175	11	233	21093	878	0.254
NMR_Kim[c]	Naked mole rat	0.0872	0.0590	**0.045**	35	2827	22241	236	**0.037**	24	110	25717	3902	**0.022**

[a] Genes under positive selection in dolphin, as identified in (Sun et al., 2013).

[b] Genes under positive selection in whale (Yim et al., 2014) were used as a negative control.

[c] Positively selected genes in naked mole rat, originally identified in (Kim et al., 2011).
